# Supplementary material for: Cost-Effective Next Generation Sequencing-Based STR Typing with Improved Analysis of Minor, Degraded and Inhibitor-Containing DNA Samples
Source: Int J Mol Sci. 2023 Feb 8;24(4):3382. doi: 10.3390/ijms24043382 (PMC9960623; doi:10.3390/ijms24043382)
Supplement: Supplementary file 1 [file ijms-24-03382-s001.zip › ijms-2127708-supplementary.pdf]

Supplementary data

Supplementary Table S1. STR profile of DNA A5 and DNA B5 of the Human Random Control DNA Panel, and of HeLa DNA, as determined by capillary electrophoresis 3
Suppl. Figures S1-S6: Sensitivity study 4
Suppl. Figure S1. Sensitivity study: Sample 1 ng, human DNA A5 4
Suppl. Figure S2. Sensitivity study: Sample 500 pg, human DNA A5 4
Suppl. Figure S3. Sensitivity study: Sample 250 pg, human DNA A5 5
Suppl. Figure S4. Sensitivity study: Sample 125 pg, human DNA A5 5
Suppl. Figure S5. Sensitivity study: Sample 62.5 pg, human DNA A5 6
Suppl. Figure S6. Sensitivity study: Sample 31.25 pg, human DNA A5 6
Suppl. Figures S7-S11: Degradation study 7
Suppl. Figure S7. Degradation study: Sample 0 mU/µL DNaseI, Hela DNA 7
Suppl. Figure S8. Degradation study: Sample 0.3 mU/µL DNaseI, Hela DNA 7
Suppl. Figure S9. Degradation study: Sample 0.6 mU/µL DNaseI, Hela DNA 8
Suppl. Figure S10. Degradation study: Sample 1.19 mU/µL DNaseI, Hela DNA 8
Suppl. Figure S11. Degradation study: Sample 2.38 mU/µL DNaseI, Hela DNA 9
Suppl. Figures S12-S17: Mixture study 9
Suppl. Figure S12. Mixtures study: Sample 500 pg DNA, human DNA B5 9
Suppl. Figure S13. Mixtures study: Sample 50:50 mixture, human DNA A5 and B5 10
Suppl. Figure S14. Mixtures study: Sample 75:25 mixture, human DNA A5 and B5 10
Suppl. Figure S15. Mixtures study: Sample 90:10 mixture, human DNA A5 and B5 11
Suppl. Figure S16. Mixtures study: Sample 95:5 mixture, human DNA A5 and B5 11
Suppl. Figure S17. Mixtures study: Sample 98:2 mixture, human DNA A5 and B5 12
Suppl. Figures S18-S35: Inhibitor study 12
Suppl. Figure S18. Inhibitor study, 30 µM hematin, human DNA A5 12
Suppl. Figure S19. Inhibitor study, 60 µM hematin, human DNA A5 13
Suppl. Figure S20. Inhibitor study, 120 µM hematin, human DNA A5 13
Suppl. Figure S21. Inhibitor study, 120 µM hematin with PowerPlex MM, human DNA A5 14
Suppl. Figure S22. Inhibitor study, 120 µM hematin with BSA, human DNA A5 14
Suppl. Figure S23. Inhibitor study, 50 µM humic acid, human DNA A5 15
Suppl. Figure S24. Inhibitor study, 100 µM humic acid, human DNA A5 15
Suppl. Figure S25. Inhibitor study, 200 µM humic acid, human DNA A5 16
Suppl. Figure S26. Inhibitor study, 200 µM humic acid with PowerPlex MM, human DNA A5 16
Suppl. Figure S27. Inhibitor study, 200 µM humic acid with BSA, human DNA A5 17

Suppl. Figure S28. Inhibitor study, 25µM melanin, human DNA A5 17
Suppl. Figure S29. Inhibitor study, 50 µM melanin, human DNA A5 18
Suppl. Figure S30. Inhibitor study, Sample 100 µM melanin, human DNA A5 18
Suppl. Figure S31. Inhibitor study, 100 µM melanin with PowerPlex MM, human DNA A5 19
Suppl. Figure S32. Inhibitor study, Sample 100 µM melanin with BSA, human DNA A5 19
Suppl. Figure S33. Inhibitor study, 200 µM indigo, human DNA A5 20
Suppl. Figure S34. Inhibitor study, 400 µM indigo, human DNA A5 20
Suppl. Figure S35. Inhibitor study, 800 µM indigo, human DNA A5 21
Suppl. Figures S36-S38: Electropherograms of DNA A5, B5, and HeLa DNA 22
Suppl. Figure S36. Electropherogram of DNA A5 (500 pg, analysed with PowerPlex ESX17) 22
Suppl. Figure S37. Electropherogram of DNA B5 (500 pg, analysed with PowerPlex ESX17) 23
Suppl. Figure S38. Electropherogram of HeLa DNA (500 pg, analysed with PowerPlex ESX17) 24
Suppl. Figure S39: Intra- and inter-locus balances of profiles obtained with maSTR assay or CE analysis of PCR inhibitor-supplemented DNA samples 25
Suppl. Figure S40: Intra-locus balance of HeLa DNA obtained with maSTR assay or CE analysis 26
Supplementary Table S2. Calculation of sequencing costs 27

Supplementary Table S1. STR profile of DNA A5 and DNA B5 of the Human Random Control DNA Panel, and of HeLa DNA, as determined by capillary electrophoresis (see Figures S36-S38).

Locus	A5	B5*	HeLa
D10S1248	14, 15	14, 16	13, 15
D12S391	20, 23	19, 22	20, 25
D16S539	12	11	9, 10
D18S51	15, 17	14, 16	16
D19S433	13, 14	13, 14.2	13, 14
D1S1656	13, 15.3	15.3, 17.3	12, 15
D21S11	29	30, 32.2	27, 28
D22S1045	15, 16	14, 15	16, 17
D2S1338	20	17, 20	17
D2S441	14, 16	11, 14	10, 11
D3S1358	16, 18	15, 17	15, 18
D8S1179	10	9, 14	12, 13
FGA	20, 22	23, 25	18, 21
SE33	29.2, 30.2	28.2, 30.2	20
TH01	9.3	6	7
vWA	17, 18	18	16, 18

*underlined: alleles shared with DNA A5

Suppl. Figures S1-S6: Sensitivity study

Suppl. Figure S1. Sensitivity study: Sample 1 ng, human DNA A5

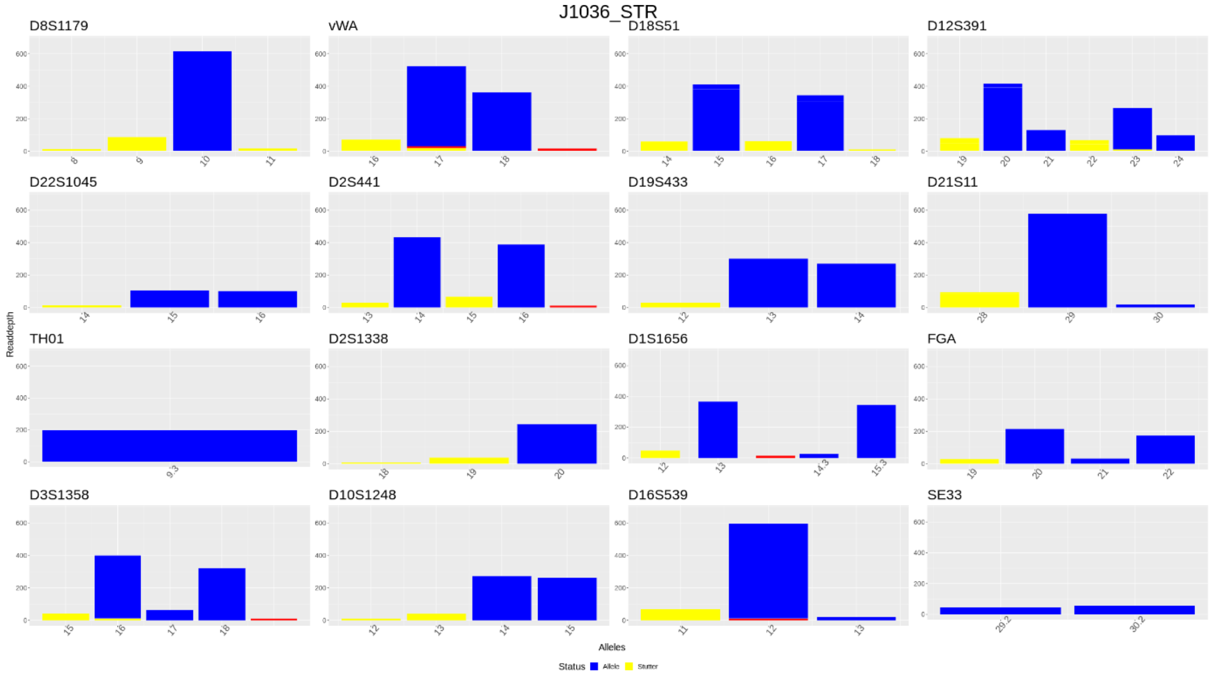

Suppl. Figure S2. Sensitivity study: Sample 500 pg, human DNA A5

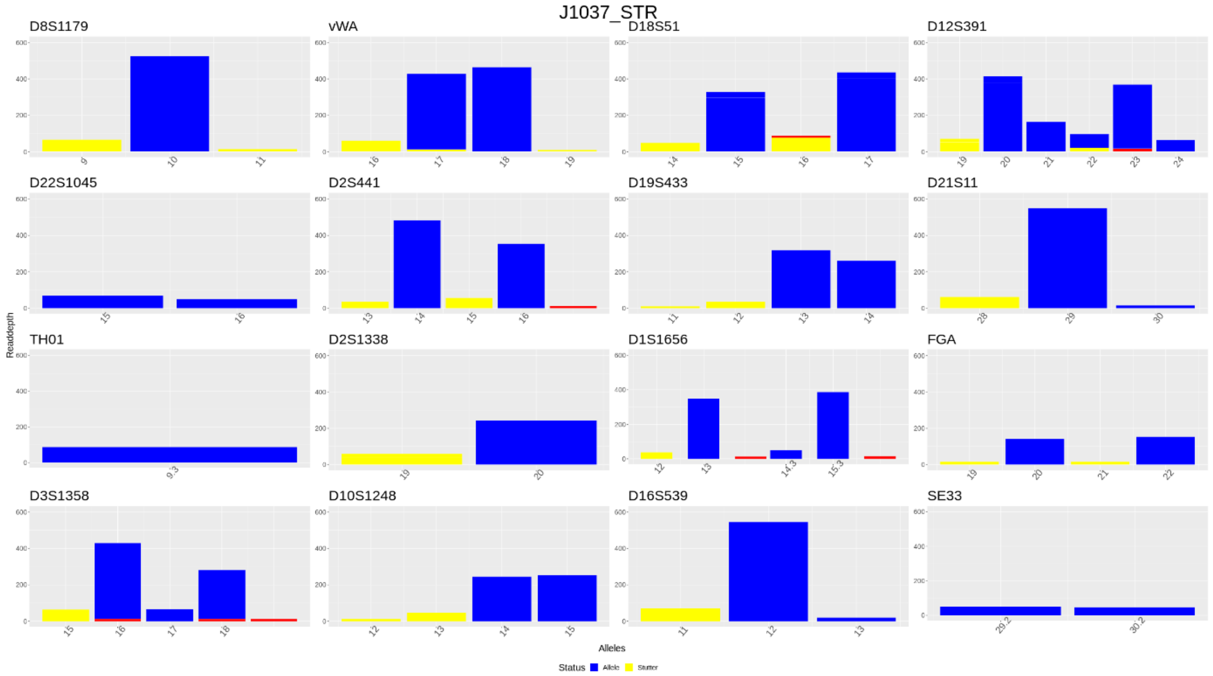

Suppl. Figure S3. Sensitivity study: Sample 250 pg, human DNA A5

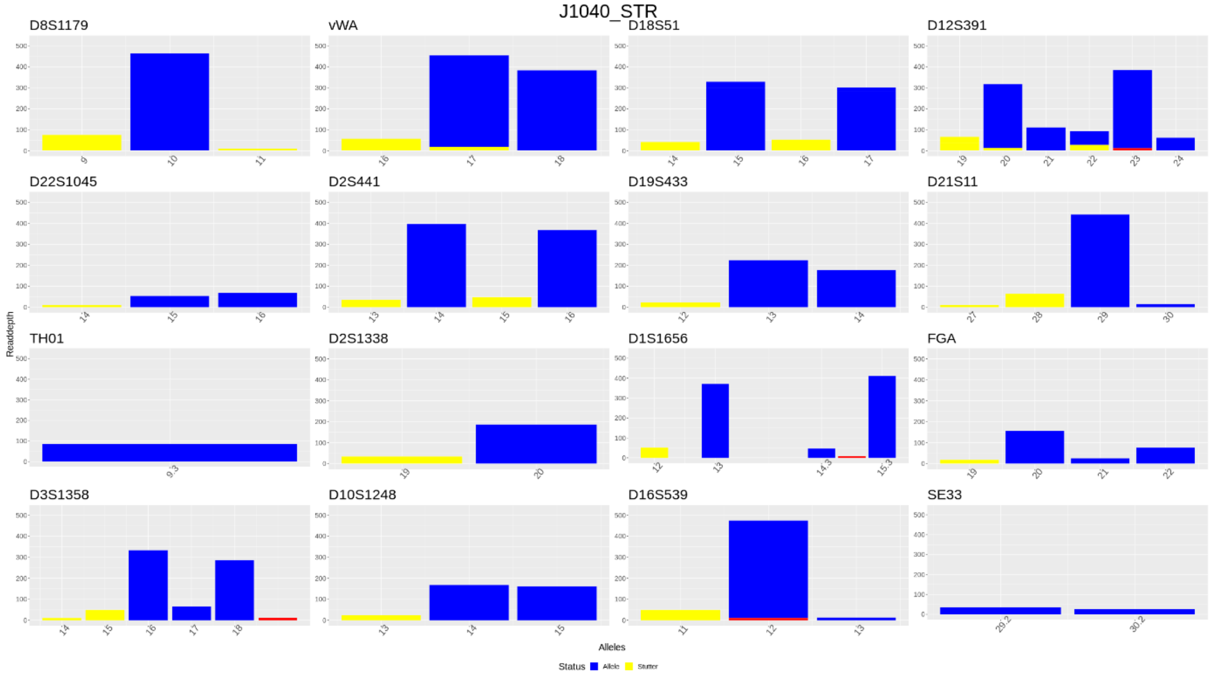

Suppl. Figure S4. Sensitivity study: Sample 125 pg, human DNA A5

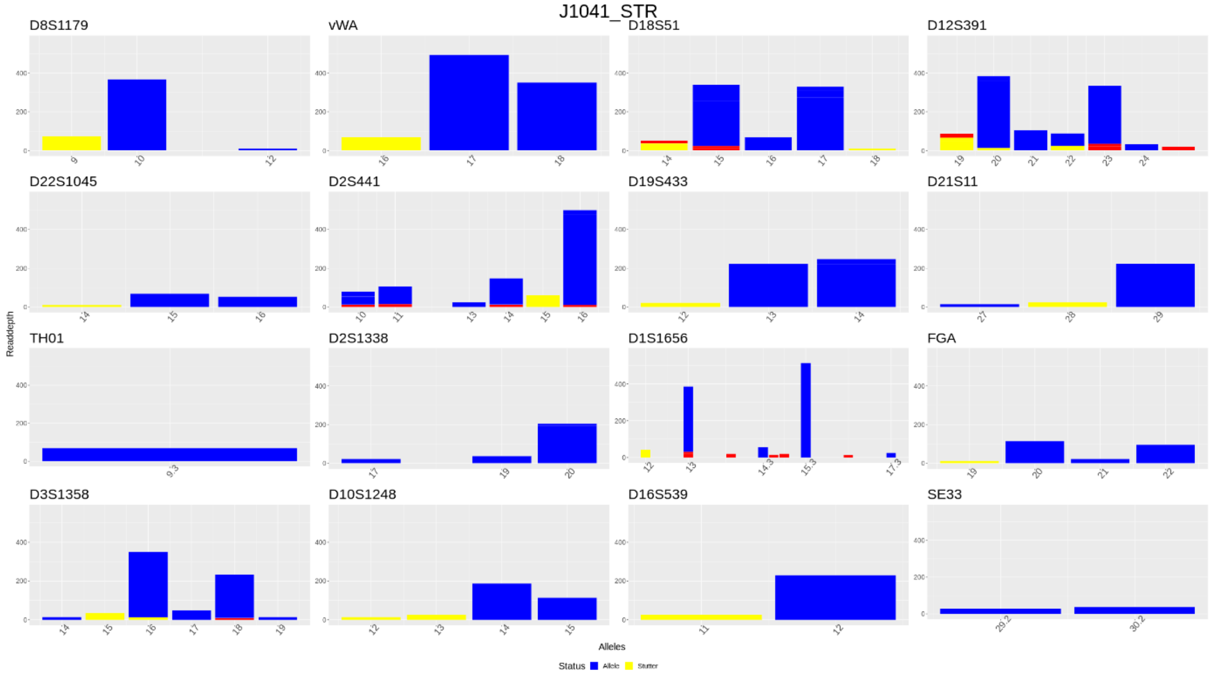

Suppl. Figure S5. **Sensitivity study: Sample 62.5 pg, human DNA A5**

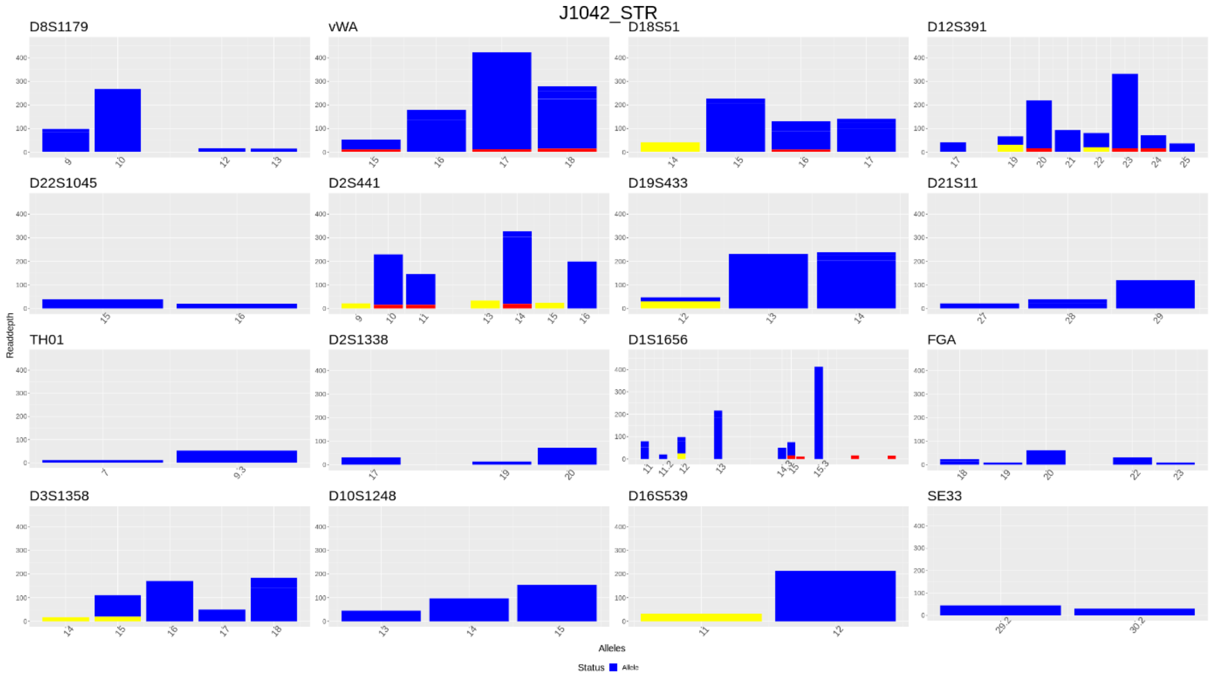

Suppl. Figure S6. Sensitivity study: Sample 31.25 pg, human DNA A5

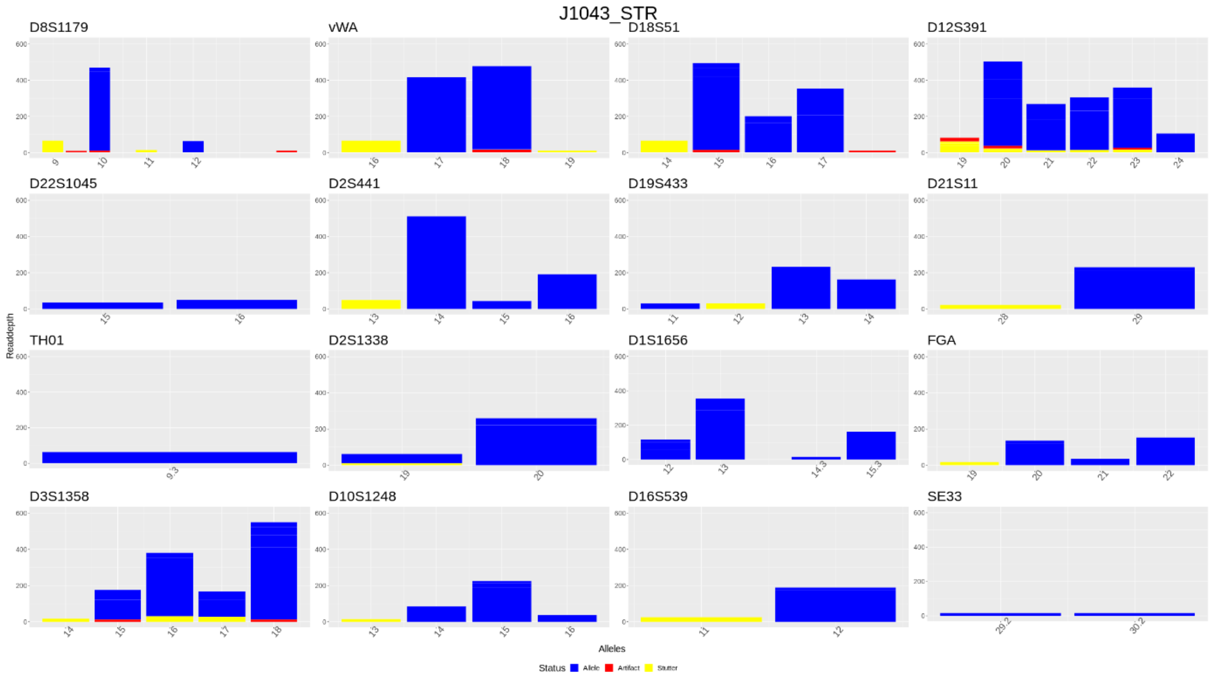

Suppl. Figures S7-S11: Degradation study

Suppl. Figure S7. Degradation study: Sample 0 mU/µL DNaseI, Hela DNA

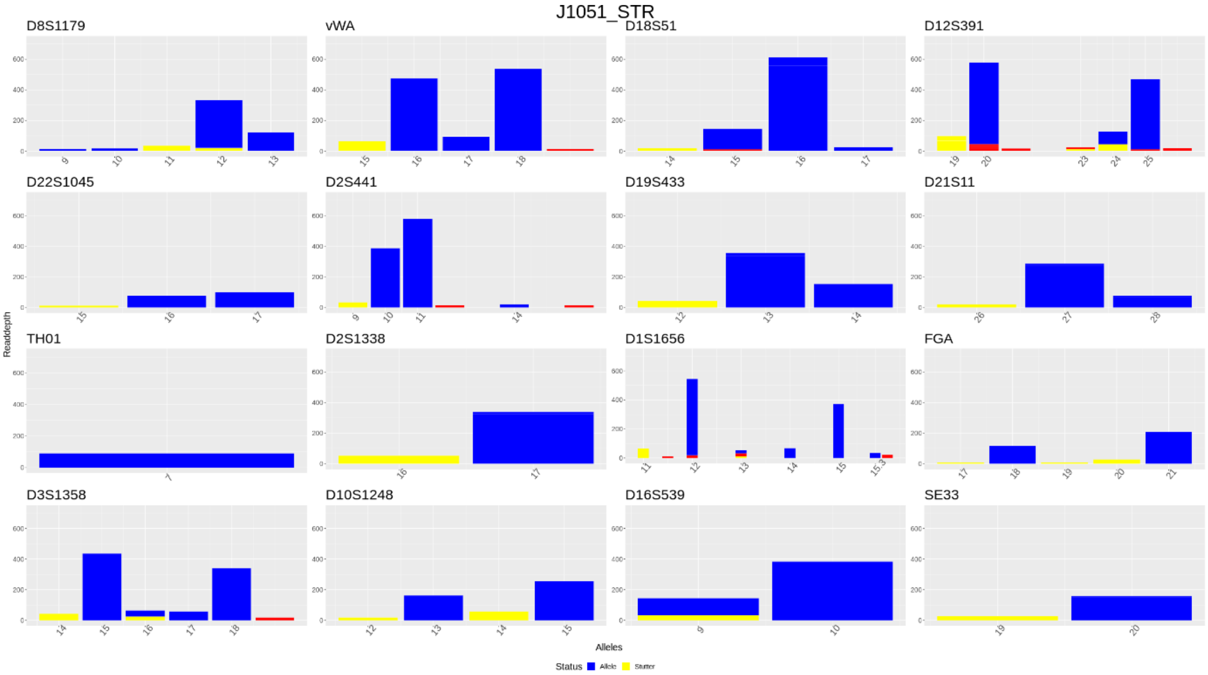

Suppl. Figure S8. Degradation study: Sample 0.3 mU/µL DNaseI, Hela DNA

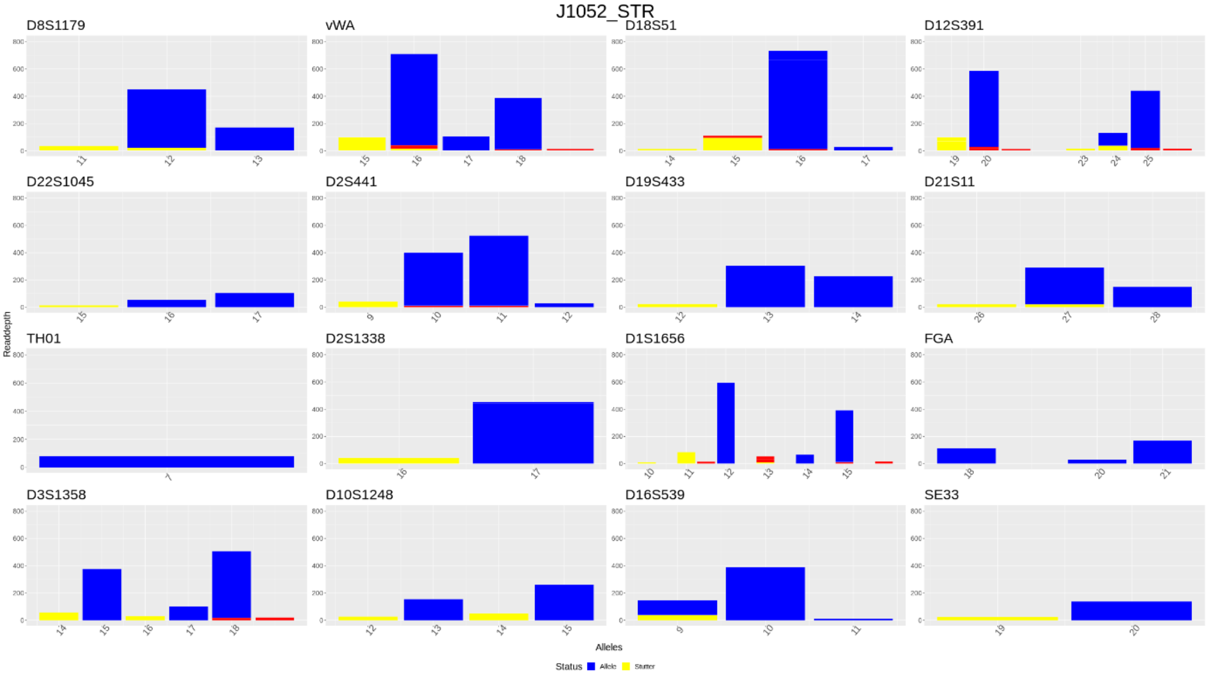

Suppl. Figure S9. Degradation study: Sample 0.6 mU/µL DNaseI, Hela DNA

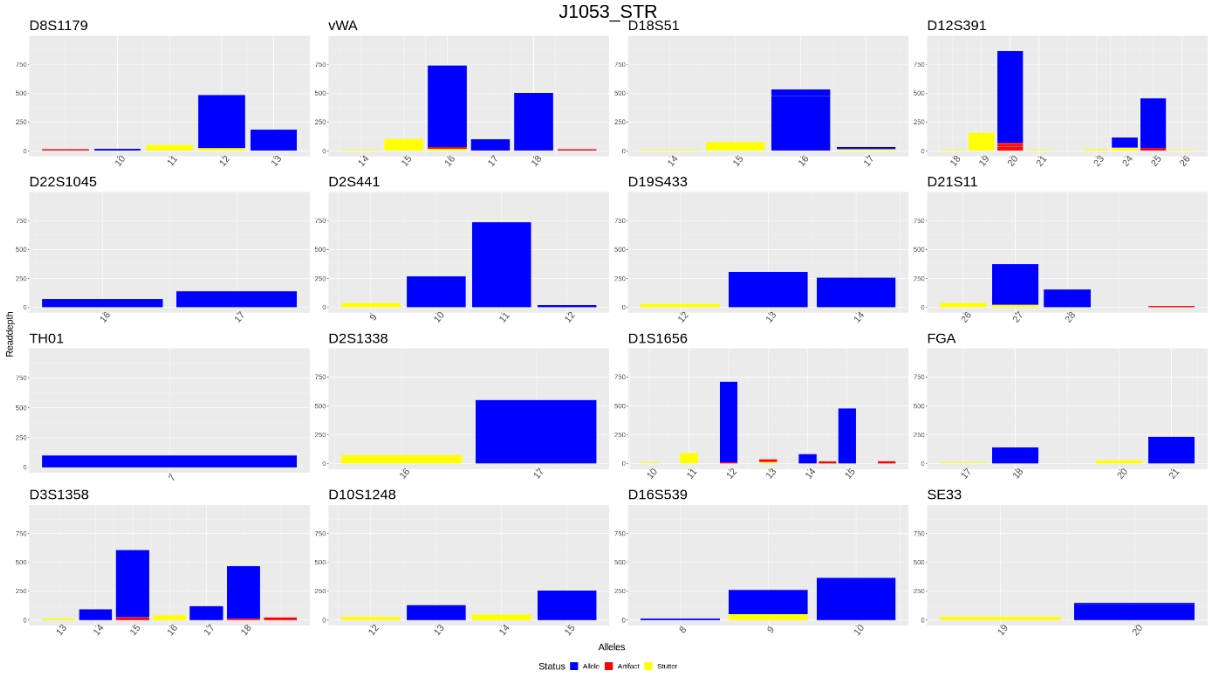

Suppl. Figure S10. Degradation study: Sample 1.19 mU/µL DNaseI, Hela DNA

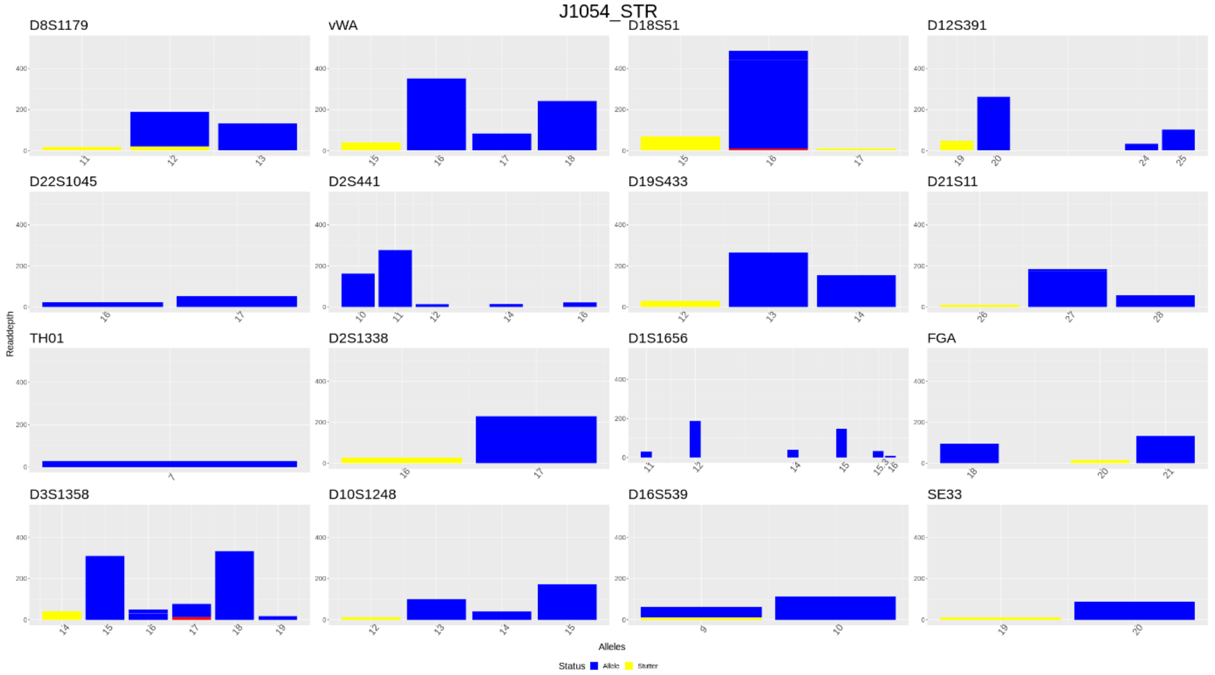

Suppl. Figure S11. Degradation study: Sample 2.38 mU/µL DNaseI, Hela DNA

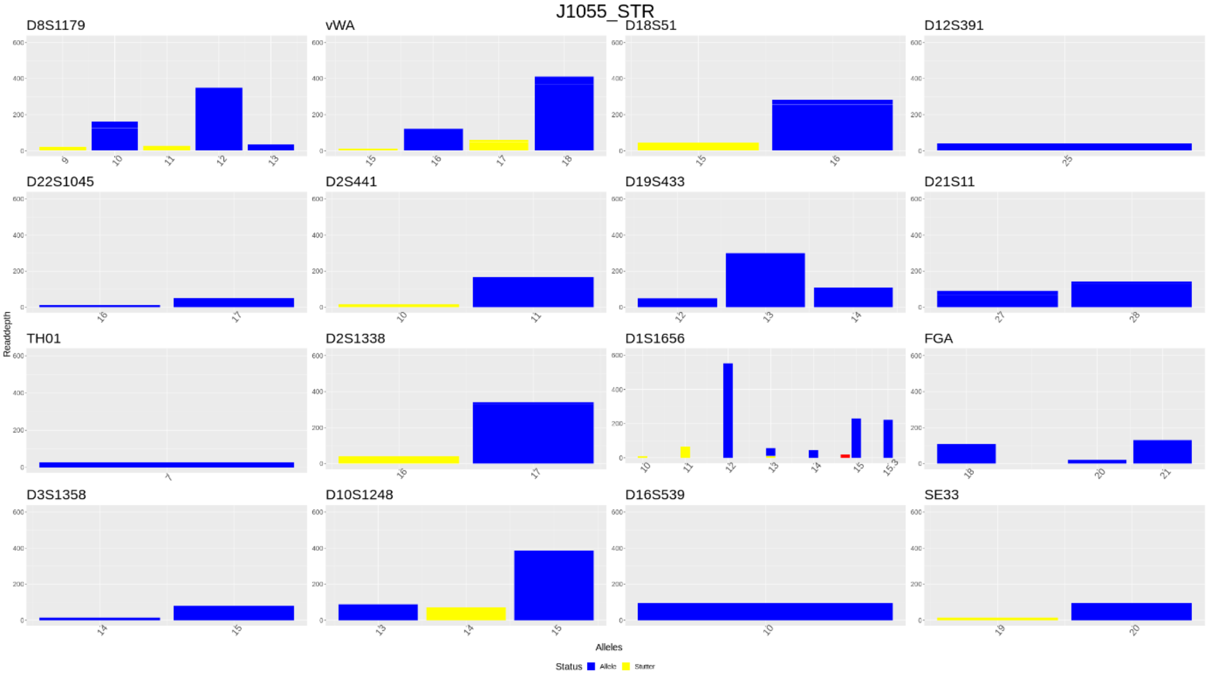

Suppl. Figures S12-S17: Mixture study

Suppl. Figure S12. Mixtures study: Sample 500 pg DNA, human DNA B5

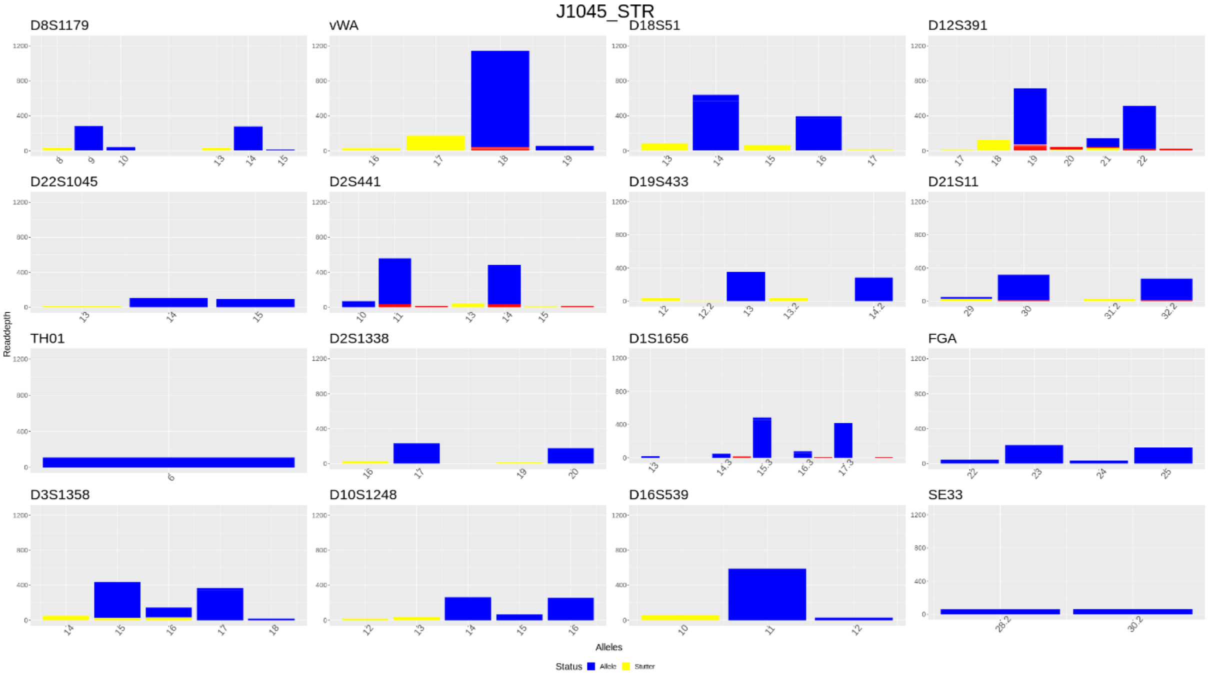

Suppl. Figure S13. Mixtures study: Sample 50:50 mixture, human DNA A5 and B5

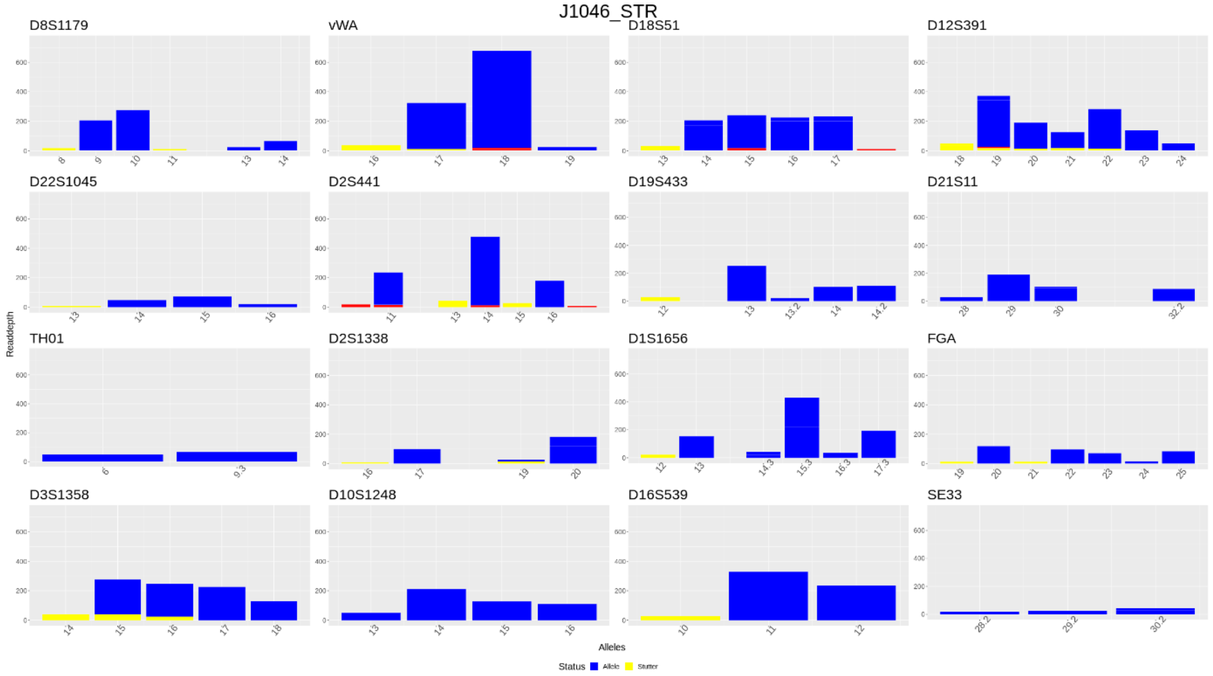

Suppl. Figure S14. Mixtures study: Sample 75:25 mixture, human DNA A5 and B5

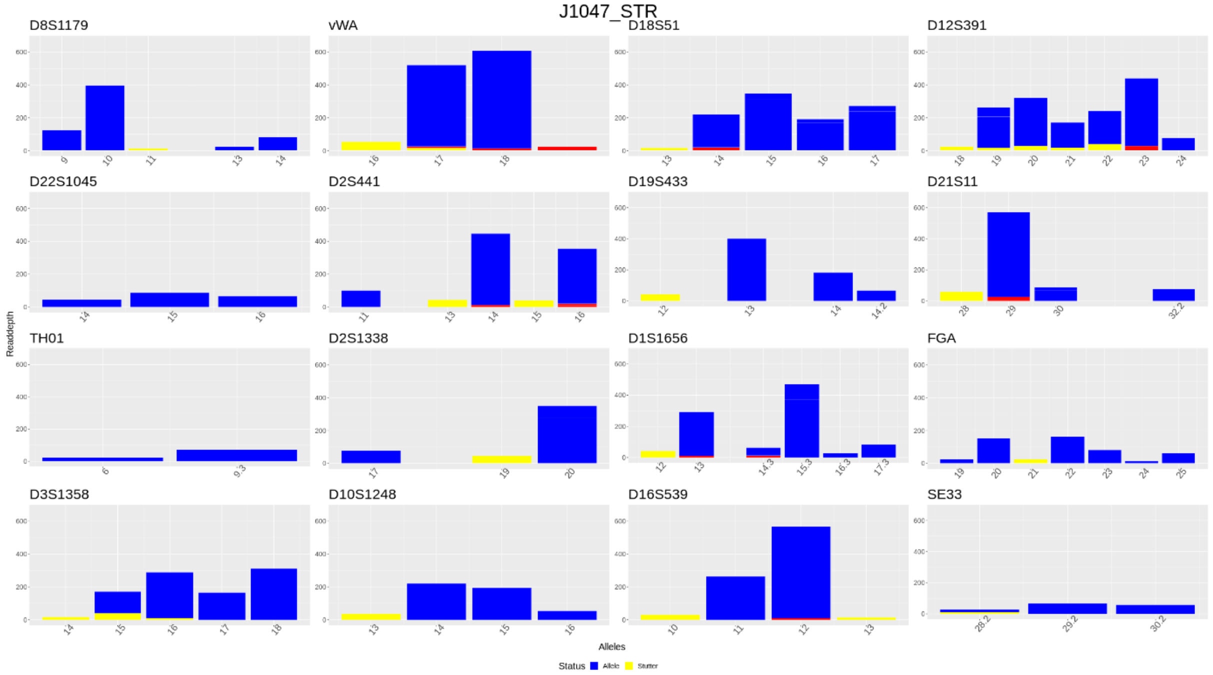

Suppl. Figure S15. Mixtures study: Sample 90:10 mixture, human DNA A5 and B5

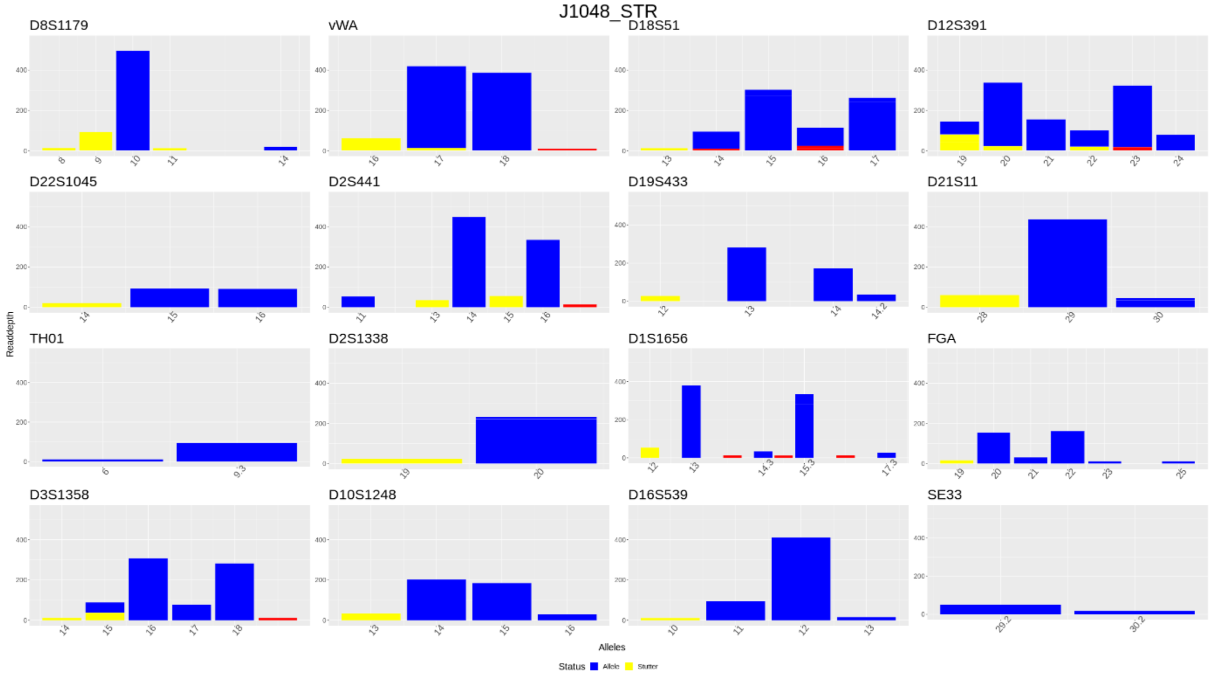

Suppl. Figure S16. Mixtures study: Sample 95:5 mixture, human DNA A5 and B5

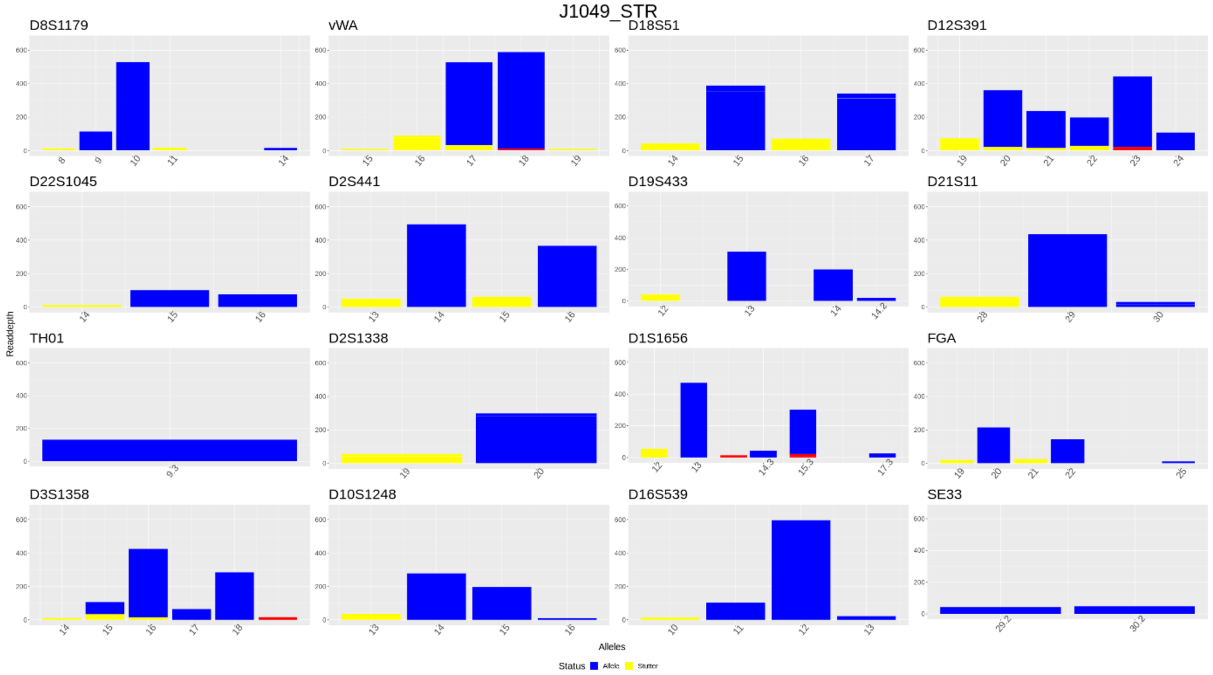

Suppl. Figure S17. Mixtures study: Sample 98:2 mixture, human DNA A5 and B5

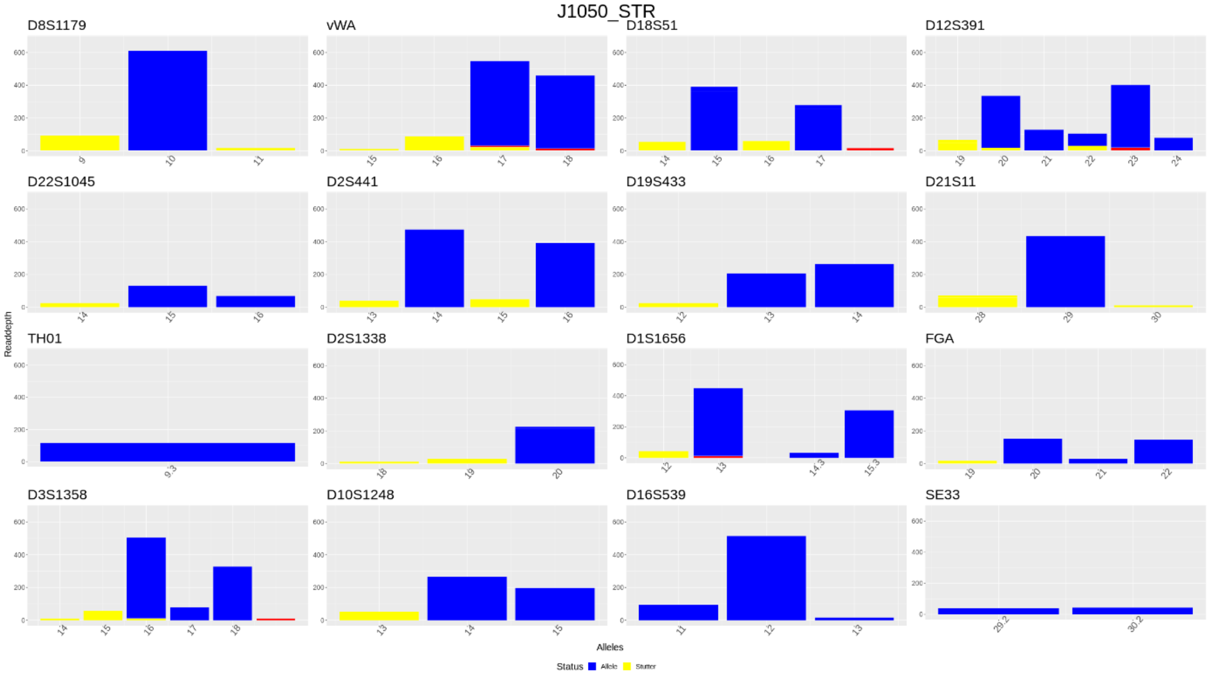

Suppl. Figures S18-S35: Inhibitor study

Suppl. Figure S18. Inhibitor study, 30 µM hematin, human DNA A5

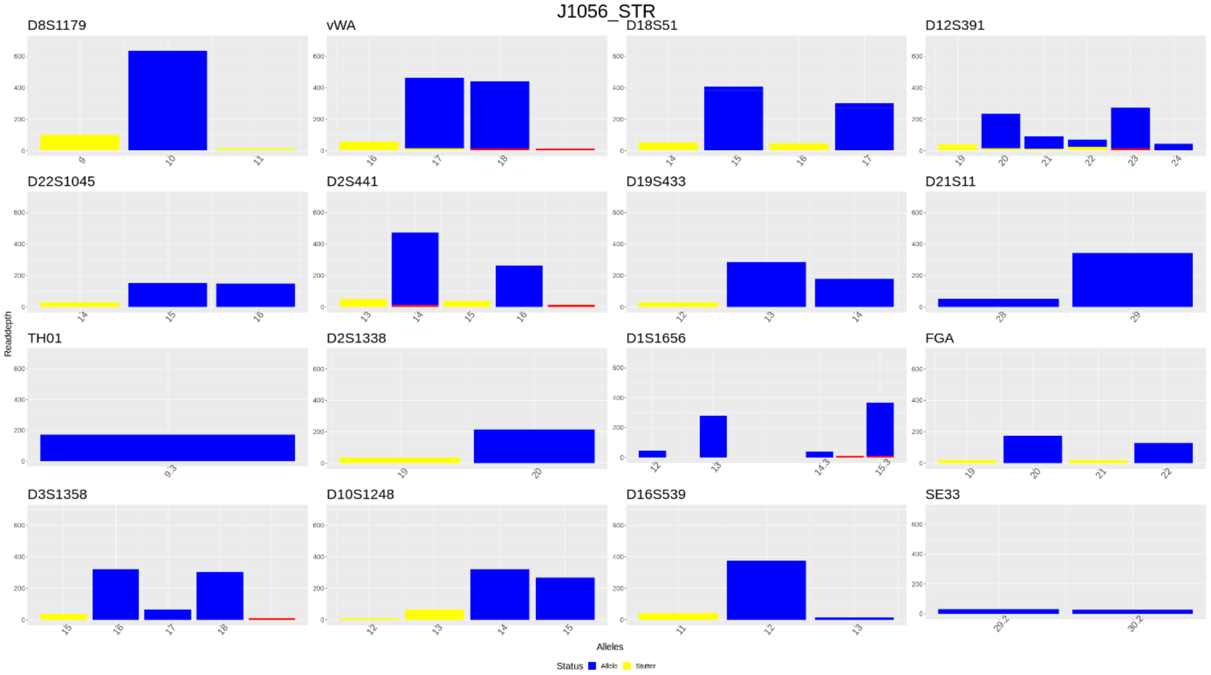

Suppl. Figure S19. Inhibitor study, 60 µM hematin, human DNA A5

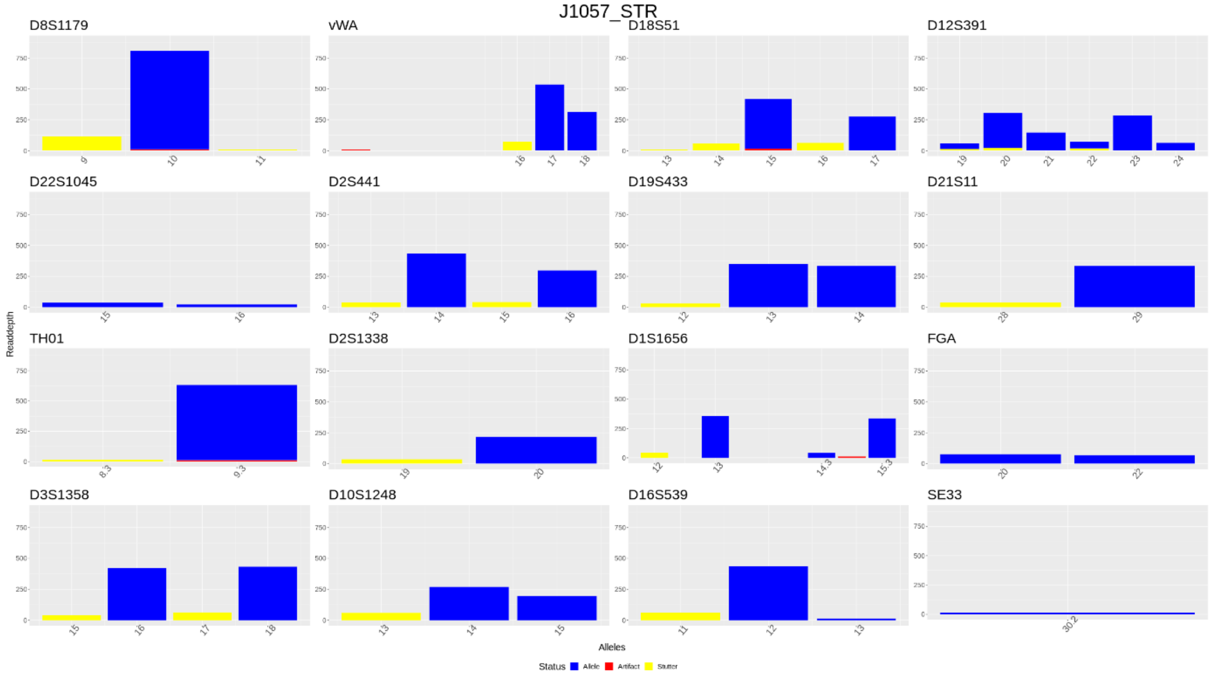

Suppl. Figure S20. Inhibitor study, 120 µM hematin, human DNA A5

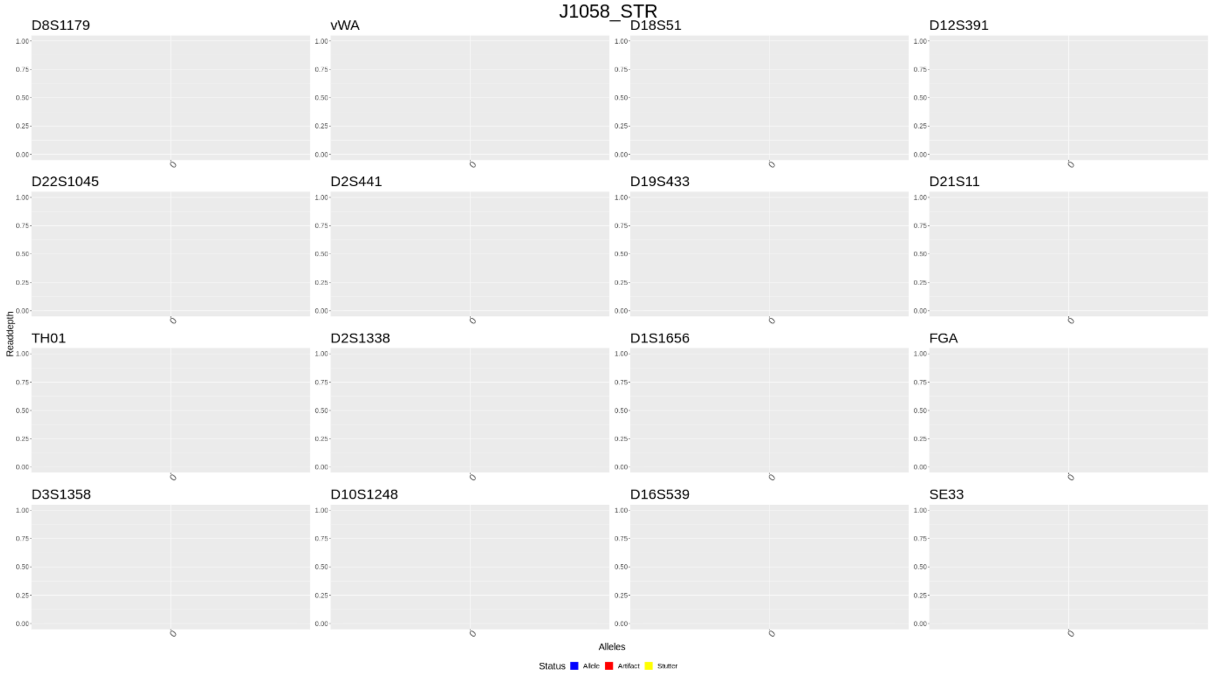

Suppl. Figure S21. Inhibitor study, 120 µM hematin with PowerPlex MM, human DNA A5

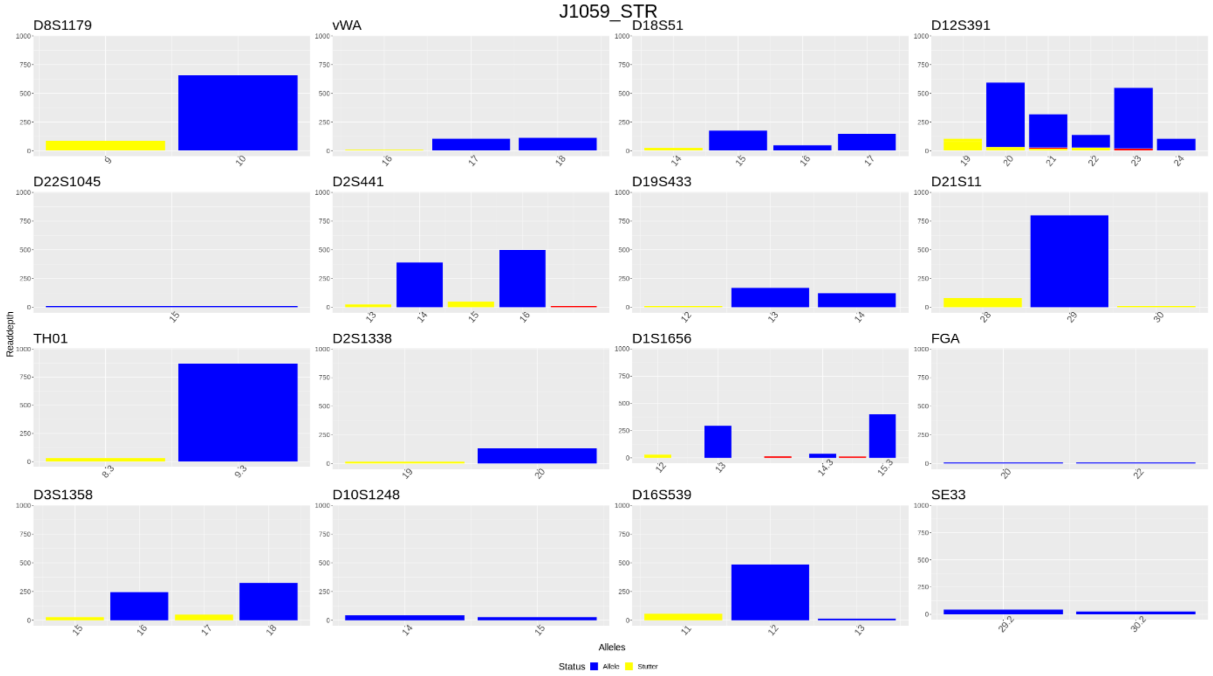

Suppl. Figure S22. Inhibitor study, 120 µM hematin with BSA, human DNA A5

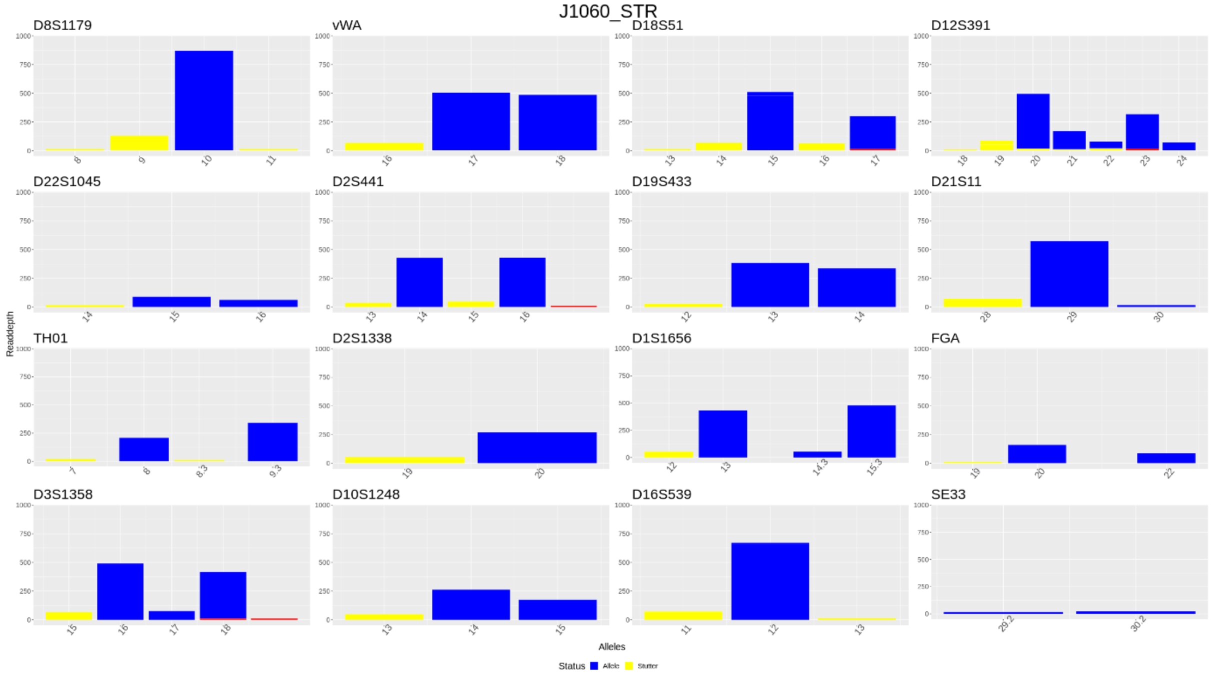

Suppl. Figure S23. Inhibitor study, 50 µM humic acid, human DNA A5

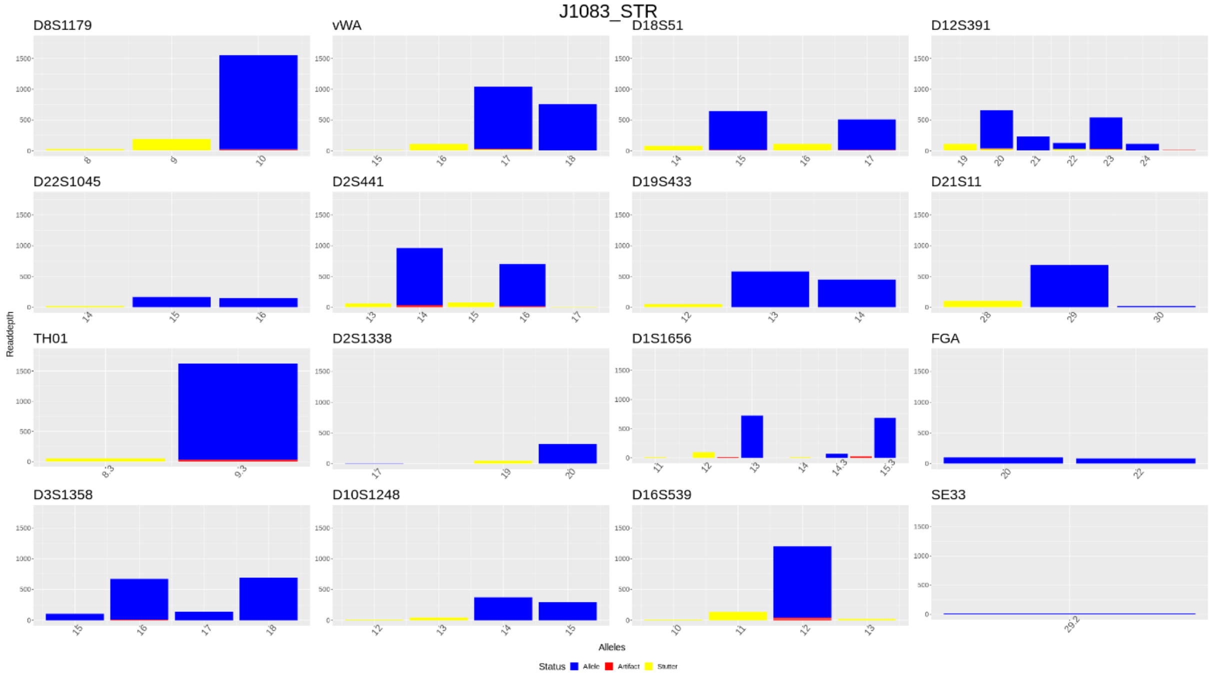

Suppl. Figure S24. Inhibitor study, 100 µM humic acid, human DNA A5

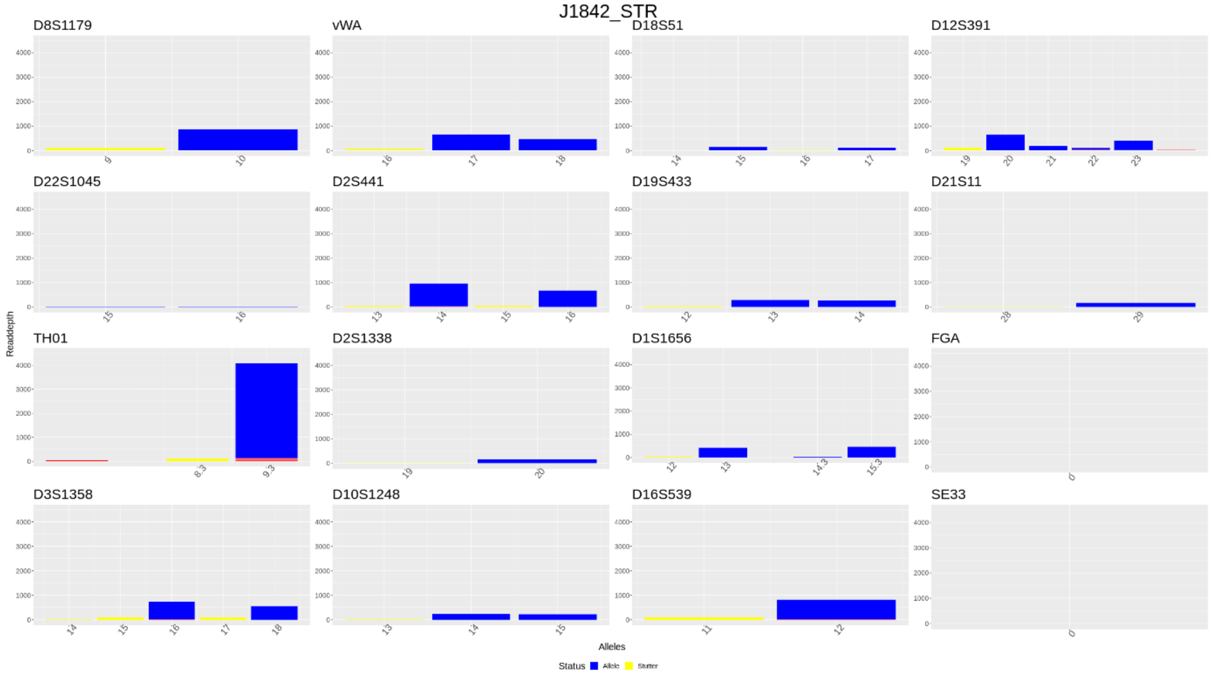

Suppl. Figure S25. Inhibitor study, 200 µM humic acid, human DNA A5

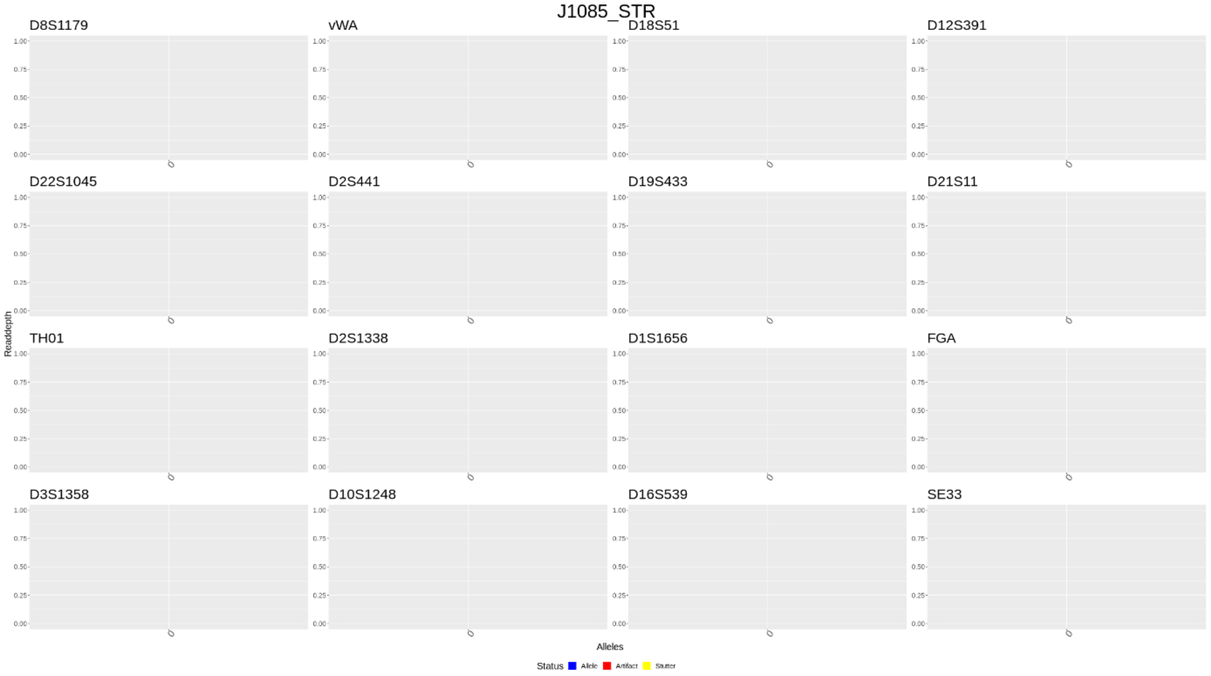

Suppl. Figure S26. Inhibitor study, 200 µM humic acid with PowerPlex MM, human DNA A5

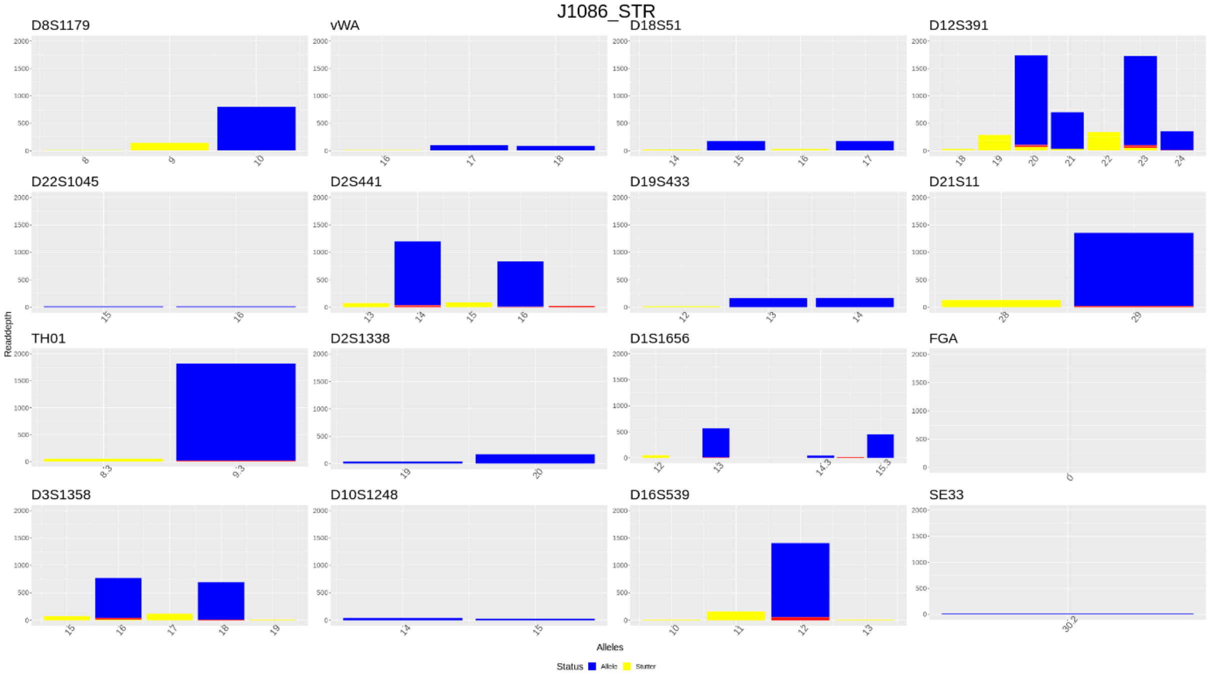

Suppl. Figure S27. Inhibitor study, 200 µM humic acid with BSA, human DNA A5

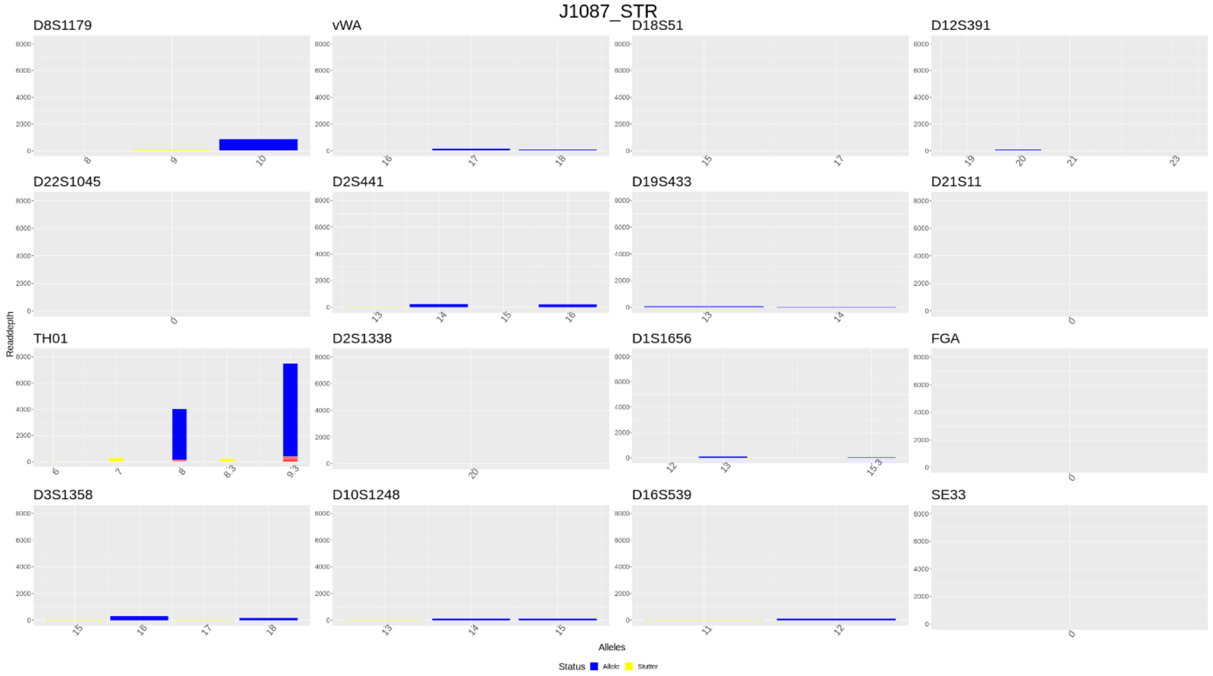

Suppl. Figure S28. Inhibitor study, 25µM melanin, human DNA A5

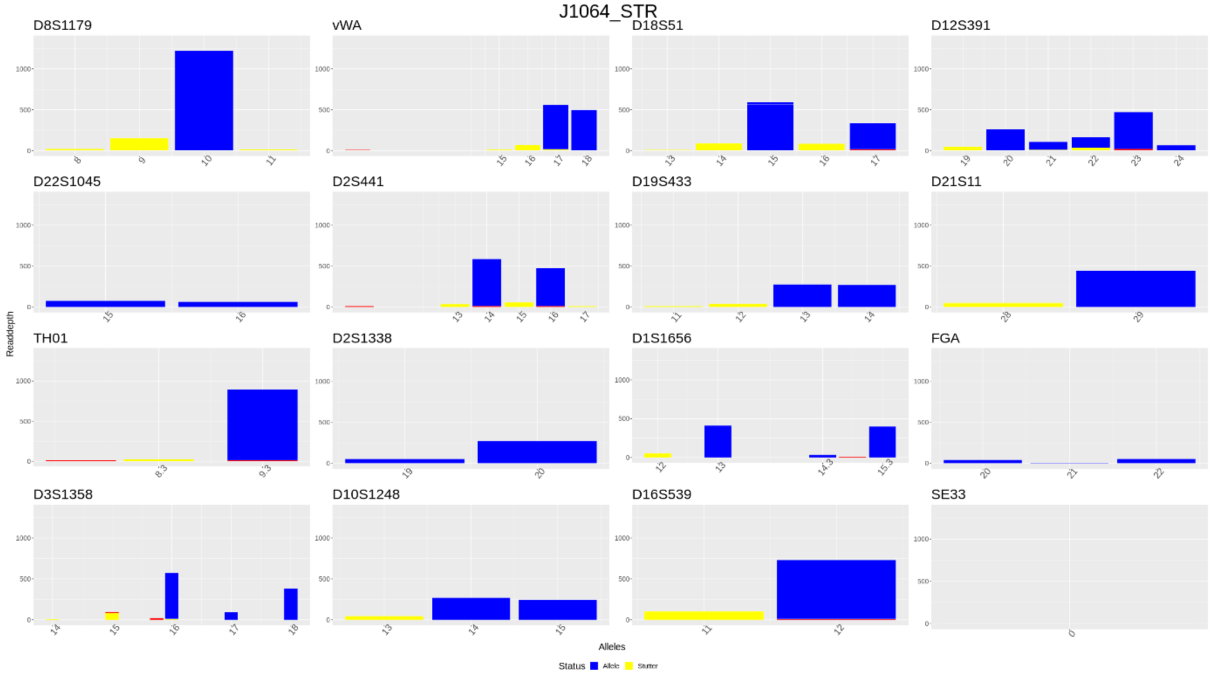

Suppl. Figure S29. Inhibitor study, 50 µM melanin, human DNA A5

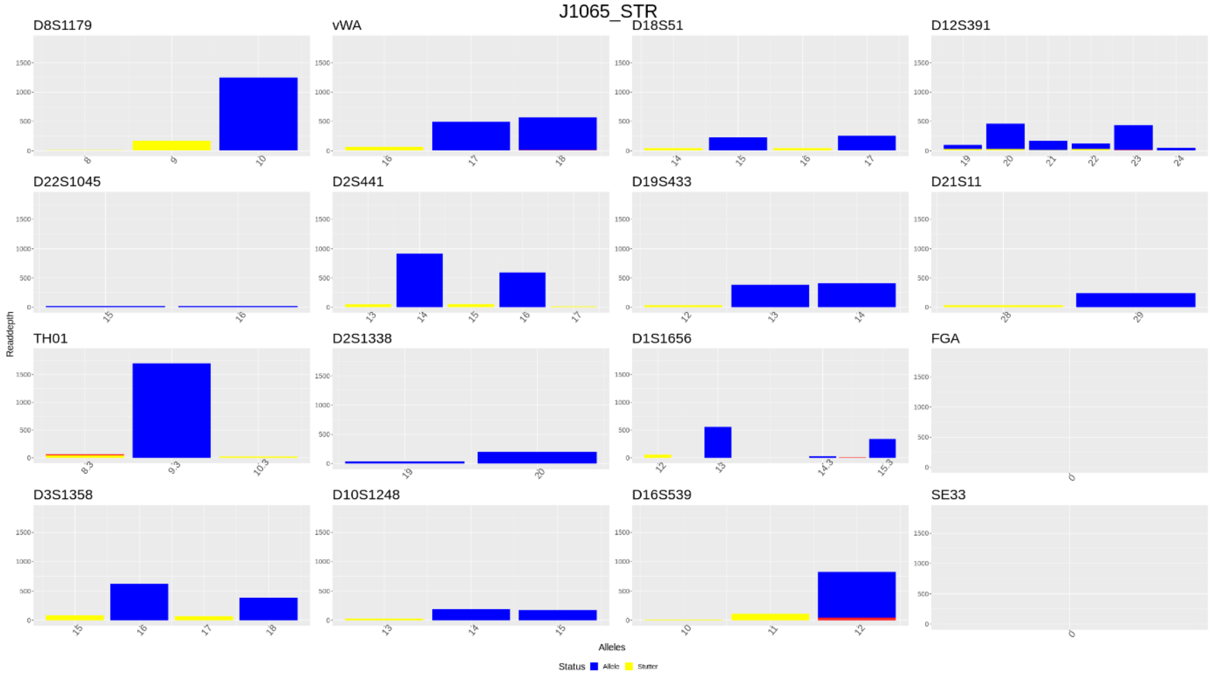

Suppl. Figure S30. Inhibitor study, Sample 100 µM melanin, human DNA A5

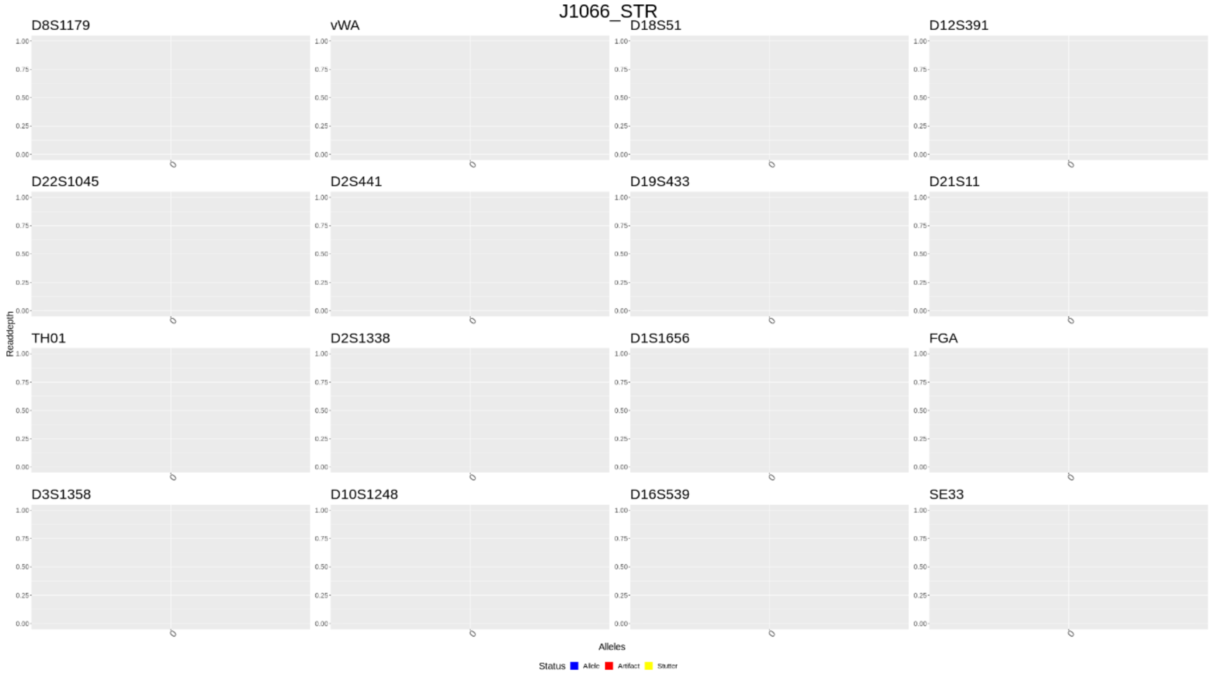

Suppl. Figure S31. Inhibitor study, 100 µM melanin with PowerPlex MM, human DNA A5

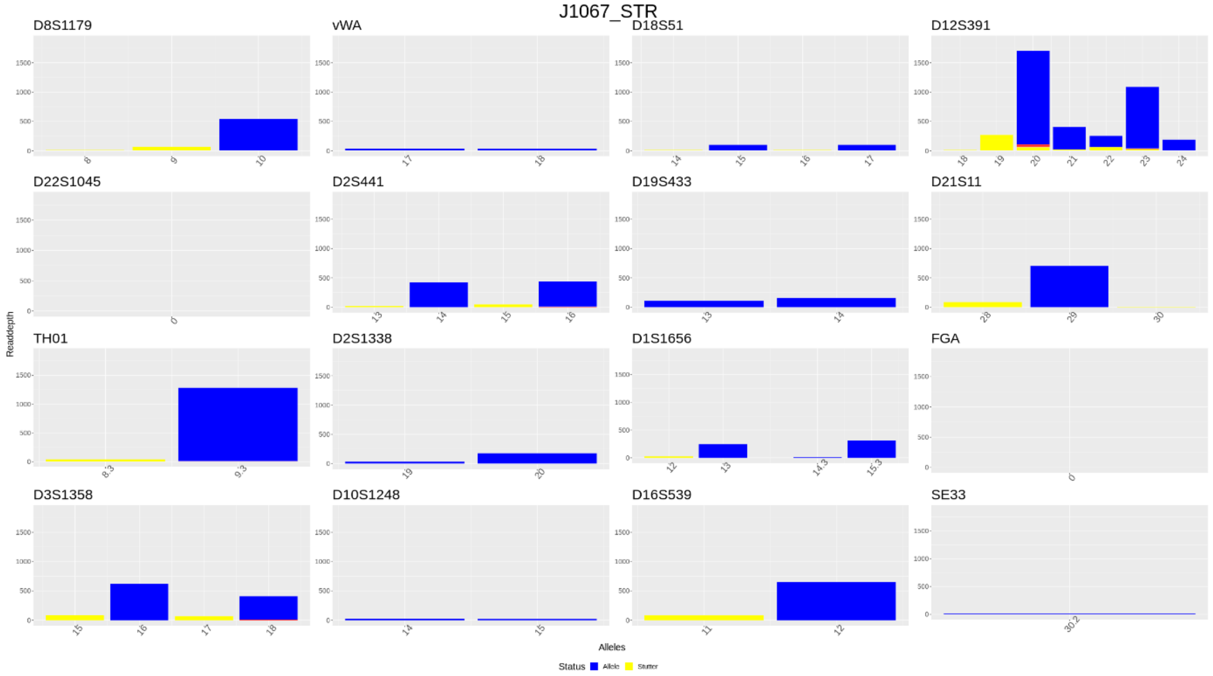

Suppl. Figure S32. Inhibitor study, Sample 100 µM melanin with BSA, human DNA A5

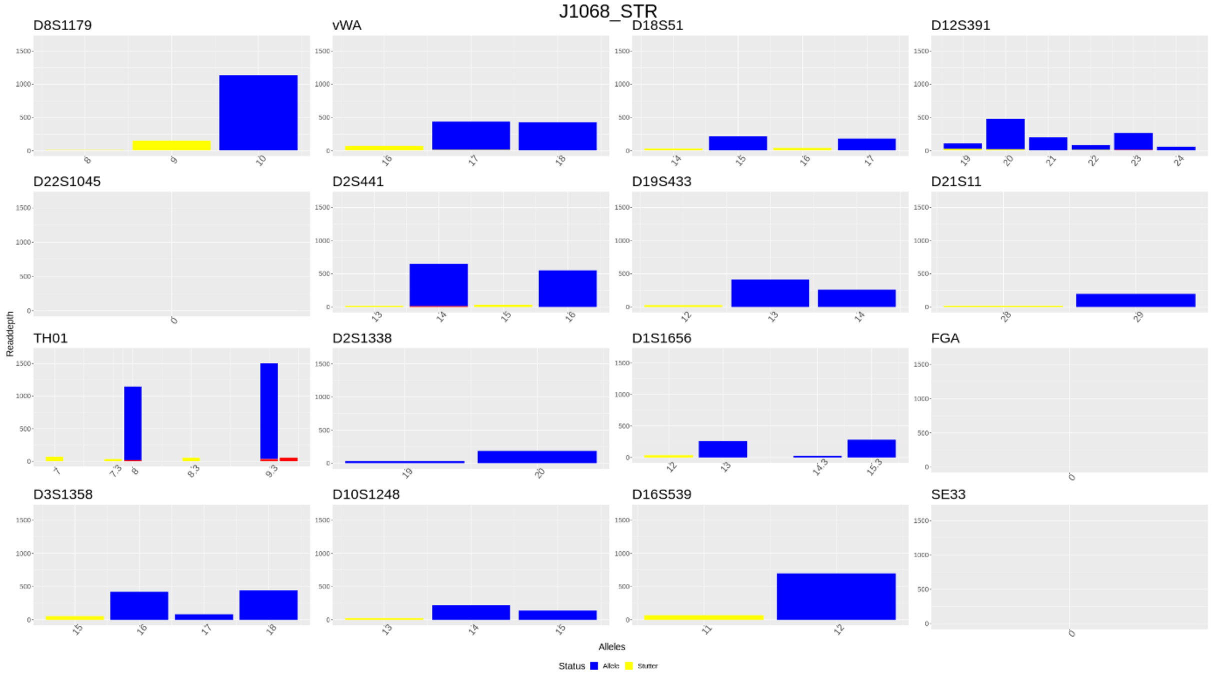

Suppl. Figure S33. Inhibitor study, 200 µM indigo, human DNA A5

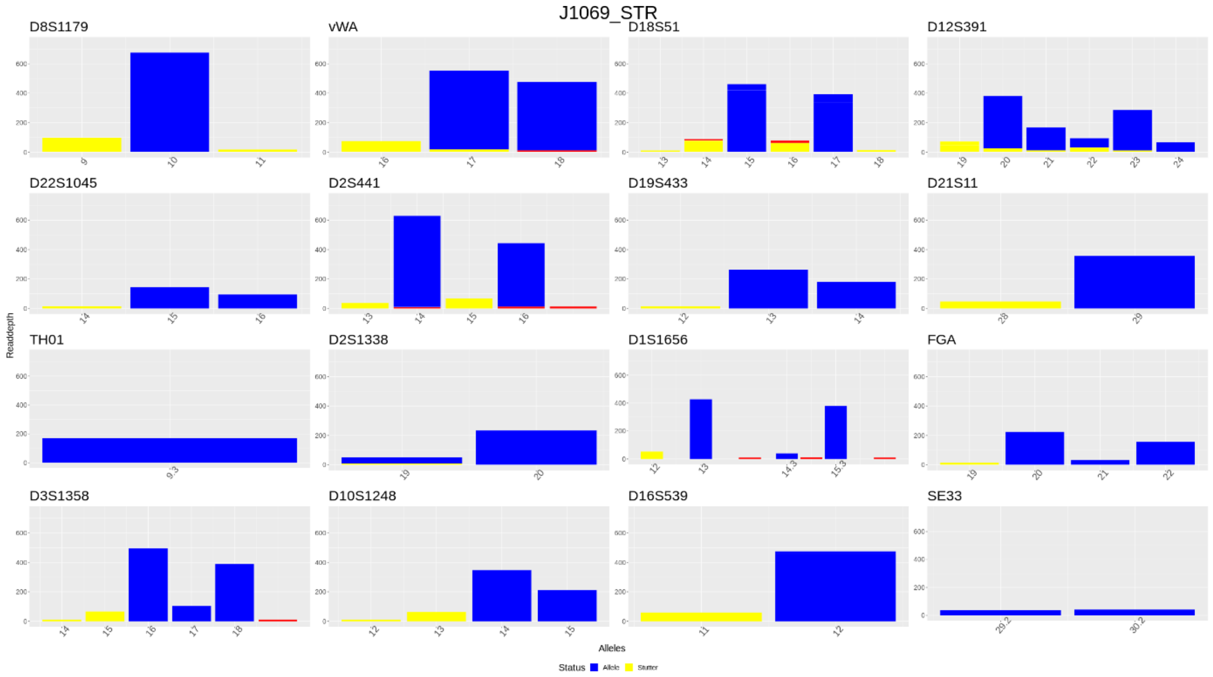

Suppl. Figure S34. Inhibitor study, 400 µM indigo, human DNA A5

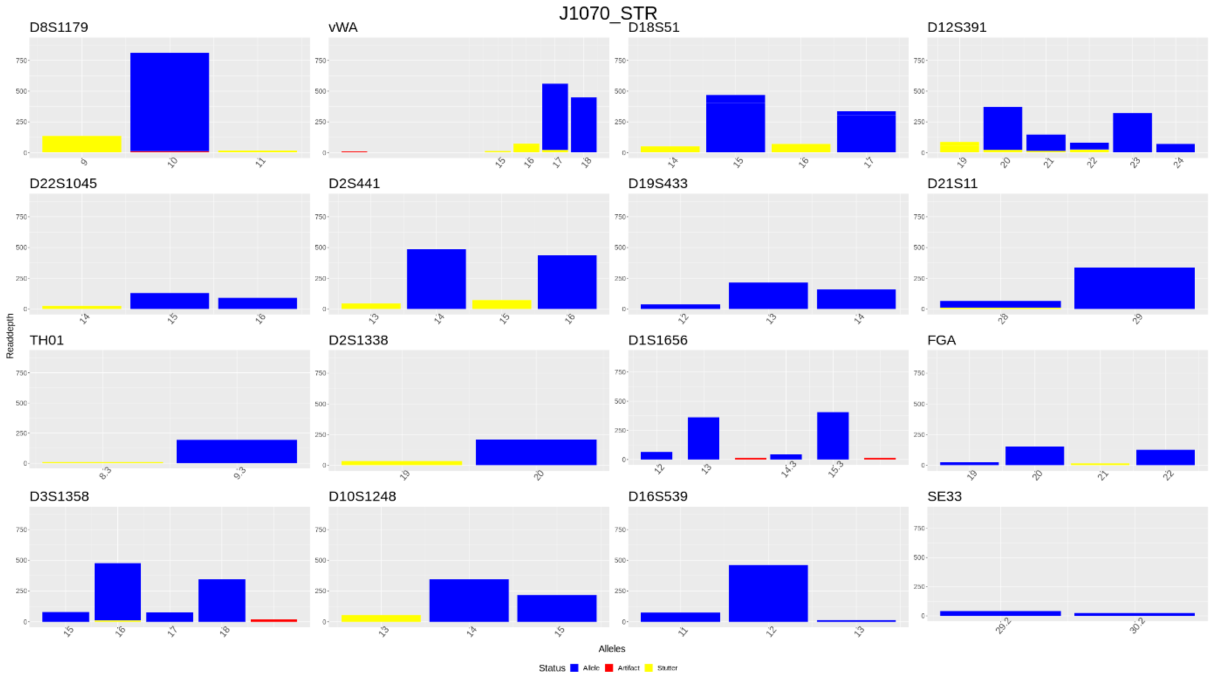

Suppl. Figure S35. Inhibitor study, 800 µM indigo, human DNA A5

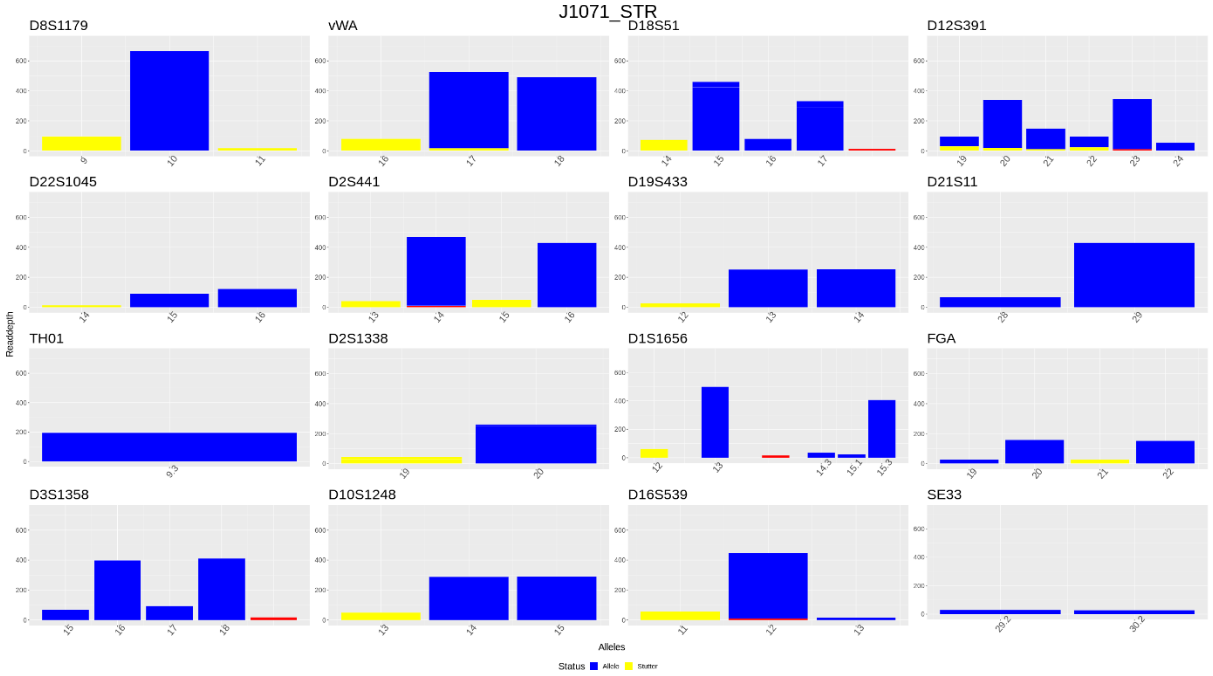

Suppl. Figures S36-S38: Electropherograms of DNA A5, B5, and HeLa DNA

Suppl. Figure S36. Electropherogram of DNA A5 (500 pg, analysed with PowerPlex ESX17)

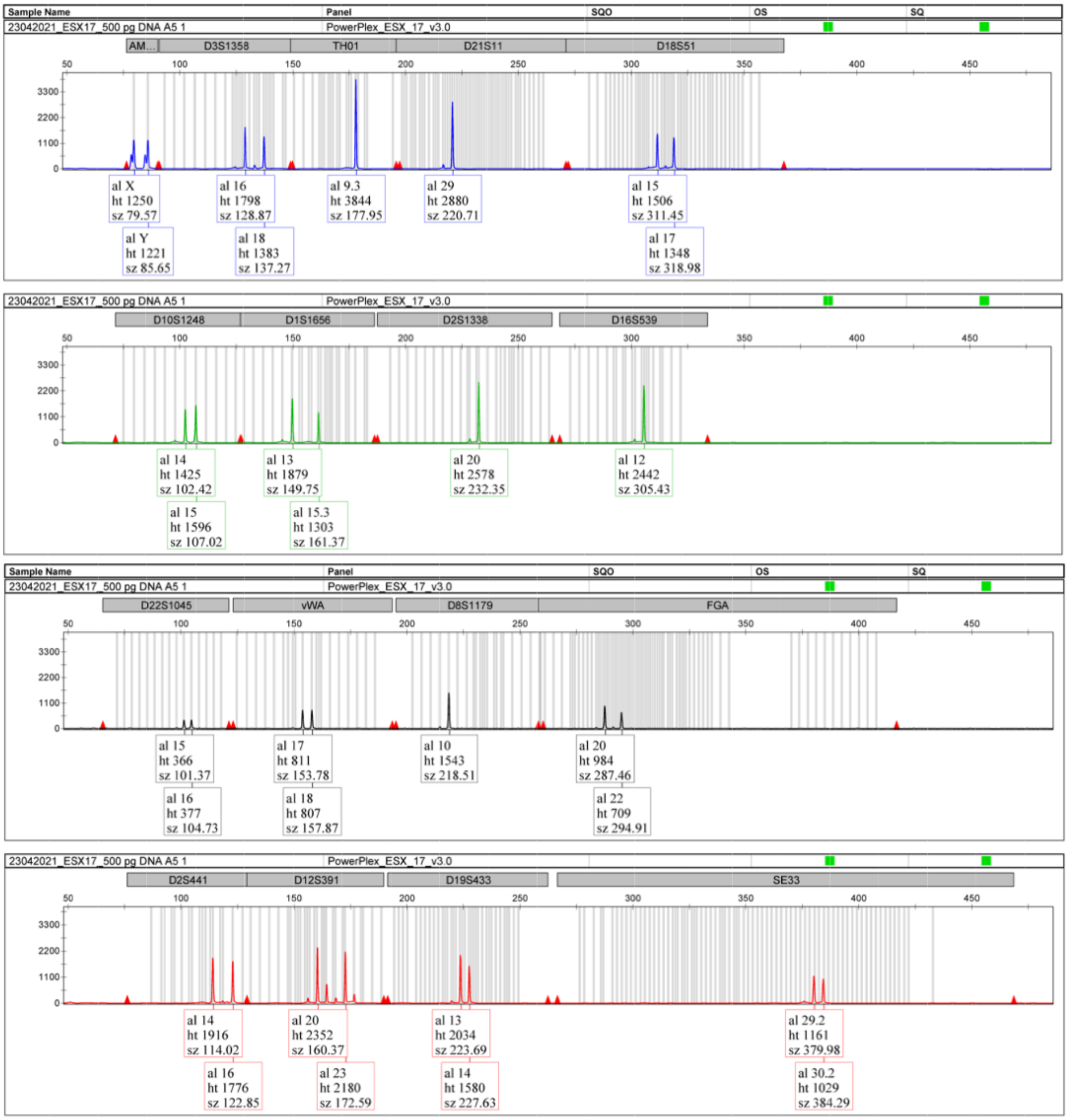

Suppl. Figure S37. Electropherogram of DNA B5 (500 pg, analysed with PowerPlex ESX17)

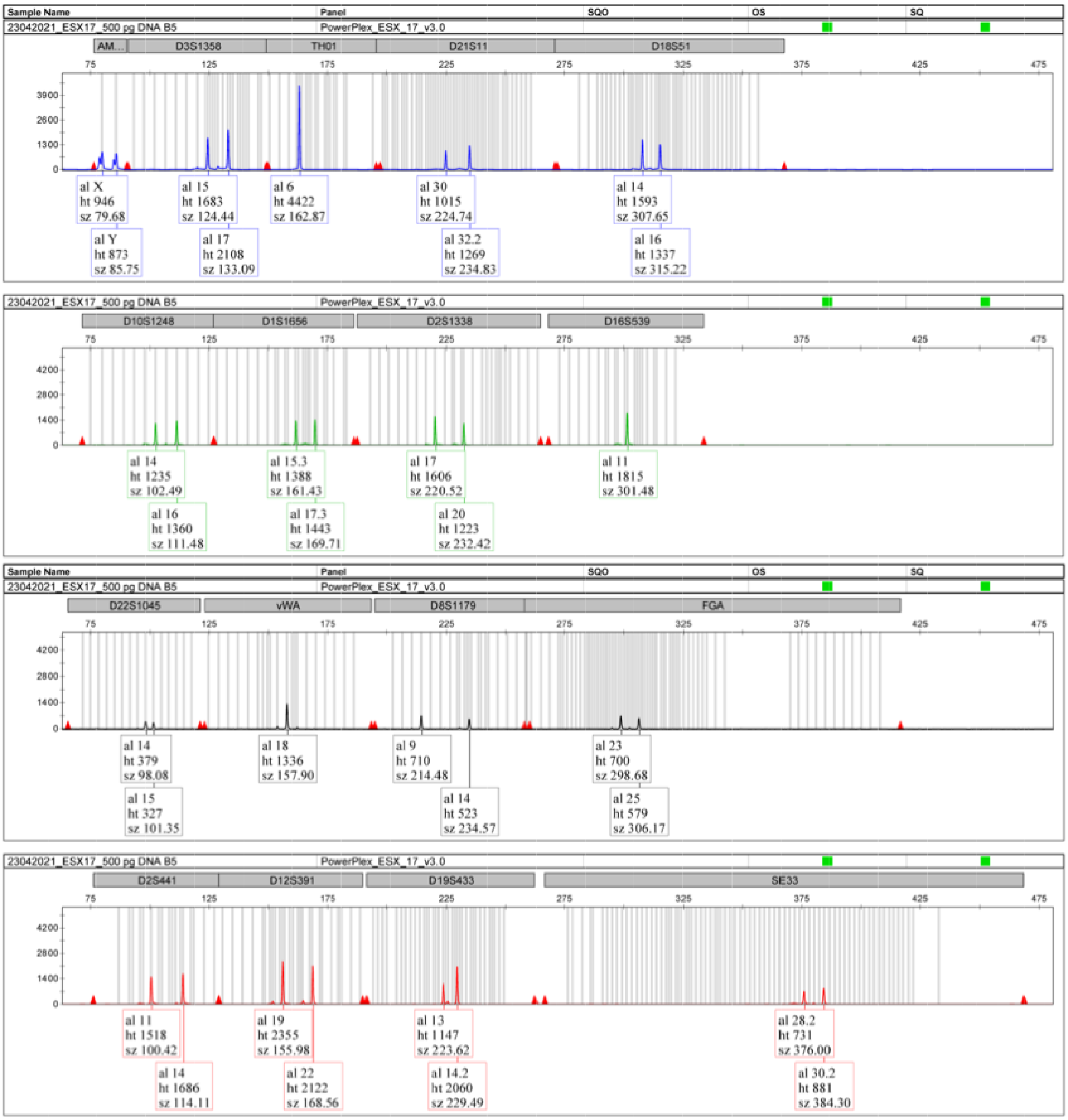

Suppl. Figure S38. Electropherogram of HeLa DNA (500 pg, analysed with PowerPlex ESX17)

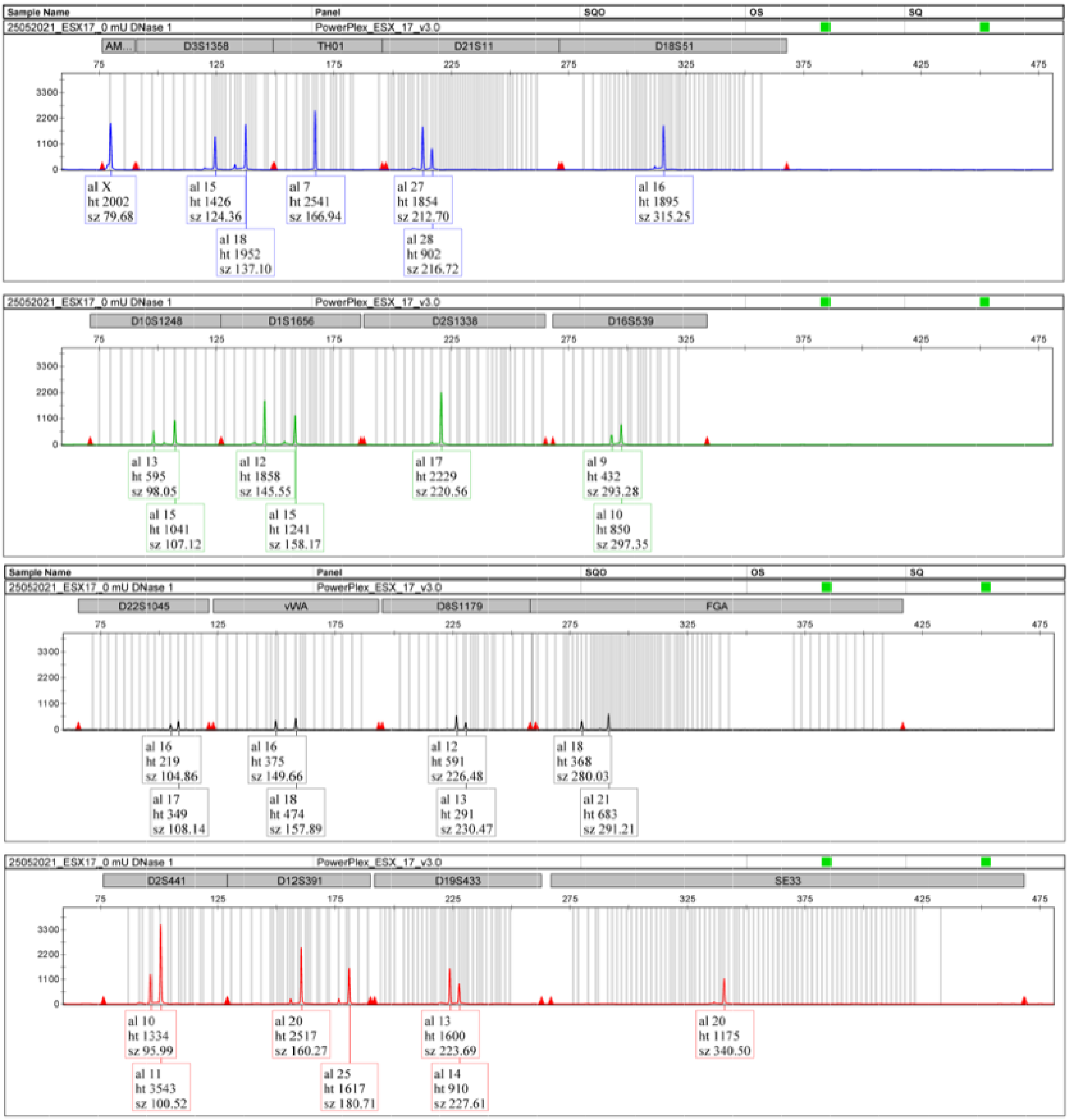

Suppl. Figure S39: Intra- and inter-locus balances of profiles obtained with maSTR assay or CE analysis of PCR inhibitor-supplemented DNA samples

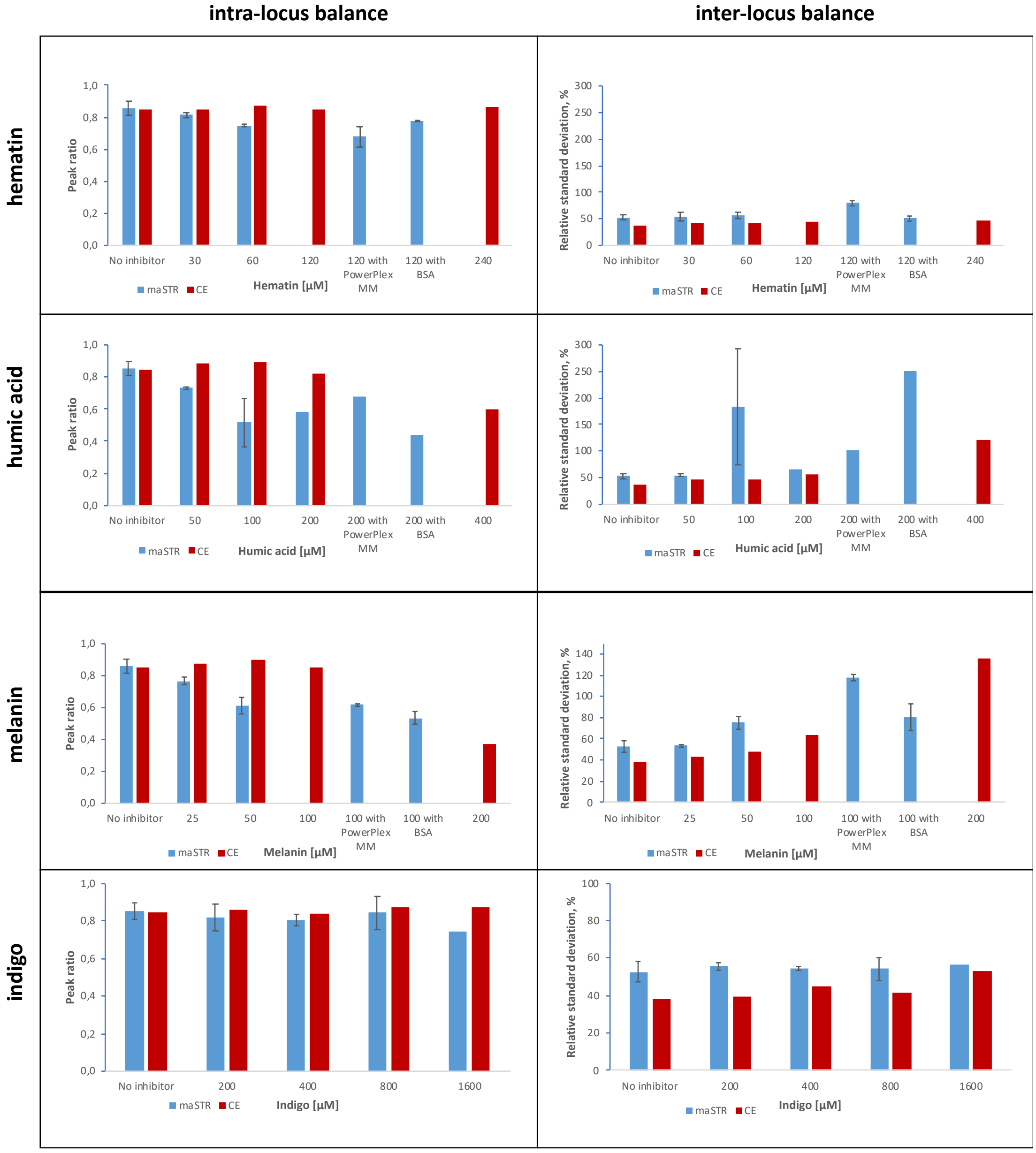

Suppl. Figure S40: Intra-locus balance of HeLa DNA obtained with maSTR assay or CE analysis

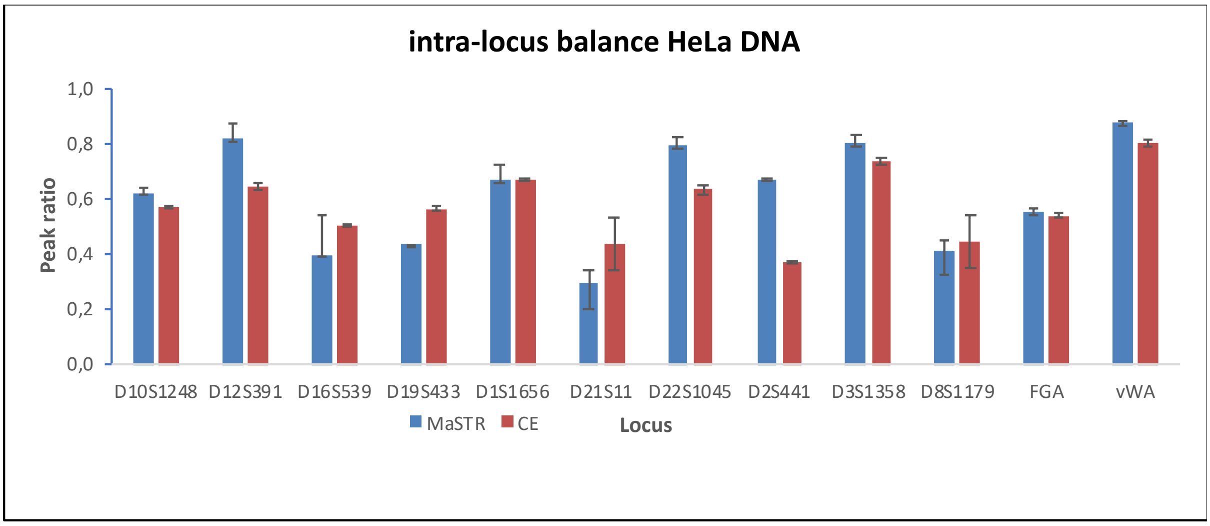

Supplementary Table S2. Calculation of sequencing costs

Library preparation costs			
**Kits**	**Cost of the kit [€]**	**Number of samples that can be processed with this amount of reagent**	**Cost per sample [€]**
**maSTR**			
QIAGEN Multiplex PCR *Plus* Kit	258	200	1,29
AMPure XP Reagent, 60 mL	960,10	789	1,22
KapaHifi HotStart ReadyMix*	450	250	1,8
Index kit	266	96	2,77
Promega ONEdsDNA quantification kit	308	500	0,62
Primers (25 nmole)	580	10.000	0,06
**Total**			**7,75**
**Verogen**			
ForenSeq DNA Signature Prep Kit	6586	96	**68,60**
**Promega**			
Promega PowerSeq® 46GY	5247	100	**52,47**

Sequencing costs					
**Workflow**	**Sequencing Kits involved**	**Cost of the kit [€]**	**Costs per sample, if to sequence 12 samples [€]**	**Costs per sample, if to sequence 32 samples [€]**	**Costs per sample, if to sequence 96 samples [€]**
maSTR	Miseq Reagent Nano Kit (500 cycles)	394	32,83	12,31	N/A
Standard Verogen	Verogen MiSeq FGx Reagent Kit	1836	153,00	57,38	19,13
Micro Verogen	Verogen MiSeq FGx Reagent Micro Kit	791	65,92	24,72	N/A
Promega PowerSeq	MiSeq Reagent Kit v3 (600 cycles)	1725	143,75	53,90	17,97

Total costs (library preparation + sequencing)				
**Workflow**	**Sequencing Kits involved**	**Total costs per sample, if to sequence 12 samples [€]**	**Total costs per sample, if to sequence 32 samples [€]**	**Total costs per sample, if to sequence 96 samples [€]**
maSTR	Miseq Reagent Nano Kit (500 cycles)	40,59	20,06	N/A
Standard Verogen	Verogen MiSeq FGx Reagent Kit	221,60	125,98	87,73
Micro Verogen	Verogen MiSeq FGx Reagent Micro Kit	134,52	93,32	N/A
Promega PowerSeq	MiSeq Reagent Kit v3 (600 cycles)	196,22	106,37	70,44
